# Ordered mesoporous nanofibers mimicking vascular bundles for lithium metal batteries

**DOI:** 10.1093/nsr/nwae081

**Published:** 2024-03-11

**Authors:** Xiaohang Zhu, Mengmeng Liu, Fanxing Bu, Xin-Yang Yue, Xiang Fei, Yong-Ning Zhou, Anqi Ju, Jianping Yang, Pengpeng Qiu, Qi Xiao, Chao Lin, Wan Jiang, Lianjun Wang, Xiaopeng Li, Wei Luo

**Affiliations:** State Key Laboratory for Modification of Chemical Fibers and Polymer Materials, Institute of Functional Materials, College of Materials Science and Engineering, Donghua University, Shanghai 201620, China; State Key Laboratory for Modification of Chemical Fibers and Polymer Materials, Institute of Functional Materials, College of Materials Science and Engineering, Donghua University, Shanghai 201620, China; Institute for Conservation of Cultural Heritage, Shanghai University, Shanghai 200444, China; Frontiers Science Center for Transformative Molecules, School of Chemistry and Chemical Engineering, Shanghai Jiao Tong University, Shanghai 200240, China; State Key Laboratory for Modification of Chemical Fibers and Polymer Materials, Institute of Functional Materials, College of Materials Science and Engineering, Donghua University, Shanghai 201620, China; Department of Materials Science, Fudan University, Shanghai 200433, China; State Key Laboratory for Modification of Chemical Fibers and Polymer Materials, Institute of Functional Materials, College of Materials Science and Engineering, Donghua University, Shanghai 201620, China; State Key Laboratory for Modification of Chemical Fibers and Polymer Materials, Institute of Functional Materials, College of Materials Science and Engineering, Donghua University, Shanghai 201620, China; State Key Laboratory for Modification of Chemical Fibers and Polymer Materials, Institute of Functional Materials, College of Materials Science and Engineering, Donghua University, Shanghai 201620, China; State Key Laboratory for Modification of Chemical Fibers and Polymer Materials, Institute of Functional Materials, College of Materials Science and Engineering, Donghua University, Shanghai 201620, China; State Key Laboratory for Modification of Chemical Fibers and Polymer Materials, Institute of Functional Materials, College of Materials Science and Engineering, Donghua University, Shanghai 201620, China; State Key Laboratory for Modification of Chemical Fibers and Polymer Materials, Institute of Functional Materials, College of Materials Science and Engineering, Donghua University, Shanghai 201620, China; State Key Laboratory for Modification of Chemical Fibers and Polymer Materials, Institute of Functional Materials, College of Materials Science and Engineering, Donghua University, Shanghai 201620, China; State Key Laboratory for Modification of Chemical Fibers and Polymer Materials, Institute of Functional Materials, College of Materials Science and Engineering, Donghua University, Shanghai 201620, China; State Key Laboratory for Modification of Chemical Fibers and Polymer Materials, Institute of Functional Materials, College of Materials Science and Engineering, Donghua University, Shanghai 201620, China

**Keywords:** ordered mesoporous nanofibers, electrospinning, oriented micelles, lithium metal batteries

## Abstract

Hierarchical self-assembly with long-range order above centimeters widely exists in nature. Mimicking similar structures to promote reaction kinetics of electrochemical energy devices is of immense interest, yet remains challenging. Here, we report a bottom-up self-assembly approach to constructing ordered mesoporous nanofibers with a structure resembling vascular bundles via electrospinning. The synthesis involves self-assembling polystyrene (PS) homopolymer, amphiphilic diblock copolymer, and precursors into supramolecular micelles. Elongational dynamics of viscoelastic micelle solution together with fast solvent evaporation during electrospinning cause simultaneous close packing and uniaxial stretching of micelles, consequently producing polymer nanofibers consisting of oriented micelles. The method is versatile for the fabrication of large-scale ordered mesoporous nanofibers with adjustable pore diameter and various compositions such as carbon, SiO_2_, TiO_2_ and WO_3_. The aligned longitudinal mesopores connected side-by-side by tiny pores offer highly exposed active sites and expedite electron/ion transport. The assembled electrodes deliver outstanding performance for lithium metal batteries.

## Teaser

This work reports a bottom-up self-assembly strategy to synthesize single-oriented ordered mesoporous nanofibers with structure mimicking vascular bundles based on electrospinning supramolecular micelle solution, showing great potential in electrochemical energy applications.

## INTRODUCTION

**Figure 1.**
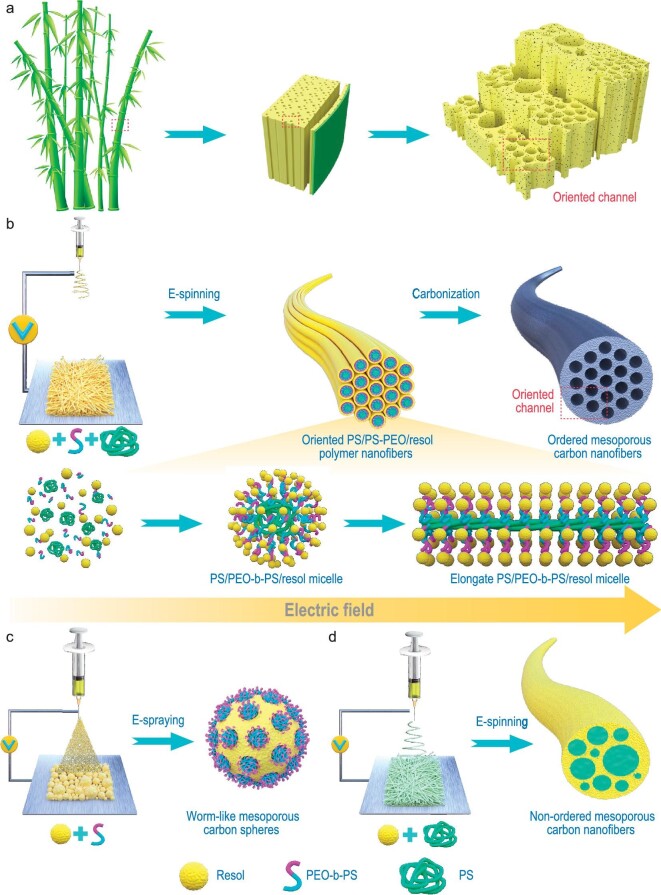
Schematic illustration of (a) the structure of bamboo [12]. (b) Electrospun synthesis of single-oriented ordered mesoporous carbon nanofibers and the electric field mediated micelle self-assembly process, which features the morphological transformation process of PS/PEO-*b*-PS/resol micelles from sphere to nanowire. Synthesis schematic for the counterparts. (c) Worm-like mesoporous carbon spheres. (d) Non-ordered mesoporous carbon nanofibers.

Earth's living creatures rely on the intake and transportation of nutrients, oxygen, and water [1,2]. Nature has developed sophisticated and efficient *in vivo* transportation systems after billions of years’ evolution [3–6]. Similar to veins in our body, vascular bundles as the key mass transportation system widely exist in terrestrial plants with lengths ranging from several centimeters to hundreds of meters [7–9]. Although there are many vascular topologies in plants, vascular bundles have two structural features, including highly oriented channels and multilevel hierarchy particularly in fast-growing plants [10,11]. Bamboo is a typical example. Its vascular bundles are composed of well-aligned longitudinal channels with a diameter of 50–200 μm running through the whole bamboo (Fig. 1a) [12,13]. The channels are connected end-to-end by uniform perforation plates, resulting in the formation of interconnected macro channels. Such an elaborate porous system boosts high-speed fluid dynamics of nutrients and water, accounting for the impressive reproducible rates of bamboo.

Similar to plants, electrochemical energy conversion and storage devices (EECSDs) such as lithium batteries and fuel cells also require efficient delivery of ions and reactants over multiple length scales [14–18]. However, transplanting the vascular structures into EECSDs faces twofold challenges. First, EECSDs demand much faster reaction kinetics and higher volumetric energy density than plants. Therefore, instead of forming macropores (>50 nm) in vascular bundles that consume excess volume, developing smaller interconnected mesopore (2–50 nm) and micropore (<2 nm) systems is favored for constructing electrode materials of EECSDs since meso-microporosity simultaneously improves ion/reactant migration kinetics and increases the number of active sites [19–22]. Nonetheless, current synthetic methodologies such as the hard template method usually result in random meso-microporosity, and consequently the formation of dead volumes and uneven ion intercalation. Second, state-of-the-art EECSDs have increasing demands for uniform and fast flow of electrolytes and electrons, particularly at high loading of active electrode materials to meet high energy goals at the cell/package level [23–26]. Thick electrodes composed of conventional nanoparticulate materials cause large diffusion impedance and inhomogeneous reactions [27,28]. Building a vascular bundle-like structure of aligned channels with nice electron conduction over the whole electrode can resolve this issue. However, to our knowledge, such nanoscale biomimicry has rarely been realized in EECSDs due to the lack of a suitable synthetic methodology.

Constructing ordered mesoporous materials via bottom-up molecular self-assembly has been under intensive study in the past decades [29,30]. Based on the controlling and co-assembling of soft amphiphilic surfactants or block copolymer micelles, ordered mesoporous materials with a rich library of regular nanoscale patterns have been fabricated [31,32]. Structural hierarchy in ordered mesoporous materials can be realized by further incorporating micropores via post-treatment [33–35]. However, most ordered mesoporous materials are in powder form, and thus pores between particles are disconnected and lack unified orientation over the long range when preparing macroscopic electrodes. To mimic vascular bundles for EECSDs, ordered mesoporous materials must possess oriented channels over at least centimeter-scale, yet remains highly challenging. The difficulty originates from the uncontrollable structure and self-assembly behaviors of the micelles, resulting in misaligned pore orientation over the long range. Moreover, the different interactions between organic/inorganic oligomers with block polymers mean those strategies only apply to certain materials, largely limiting their universality.

Here, we propose an electrospun bottom-up micelle self-assembly strategy to synthesize single-oriented ordered mesoporous nanofibers, in which the parallel mesopores are connected side-by-side with tiny channels. The synthesis involves the poly(ethylene oxide)-*b*-polystyrene (PEO-*b*-PS) block copolymer micelle as a template and polystyrene (PS) homopolymer as a pore expander. In contrast to the conventional evaporation-induced self-assembly (EISA) process, aggregated polystyrene chains are stretched into nanowires during electrospinning, which drives the structural reconstruction of the PEO-*b*-PS/precursor micelle. Well-aligned parallel mesopores over centimeter-scale can be formed in nanofibers after removing the micelle template. This approach allows large-scale fabrication of free-standing membranes, and can be generalized to fabricate ordered mesoporous nanofibers of various compositions such as carbon, SiO_2_, TiO_2_, and WO_3_.

## RESULTS AND DISCUSSION

### Synthesis and characterization of ordered mesoporous nanofibers

**Figure 2.**
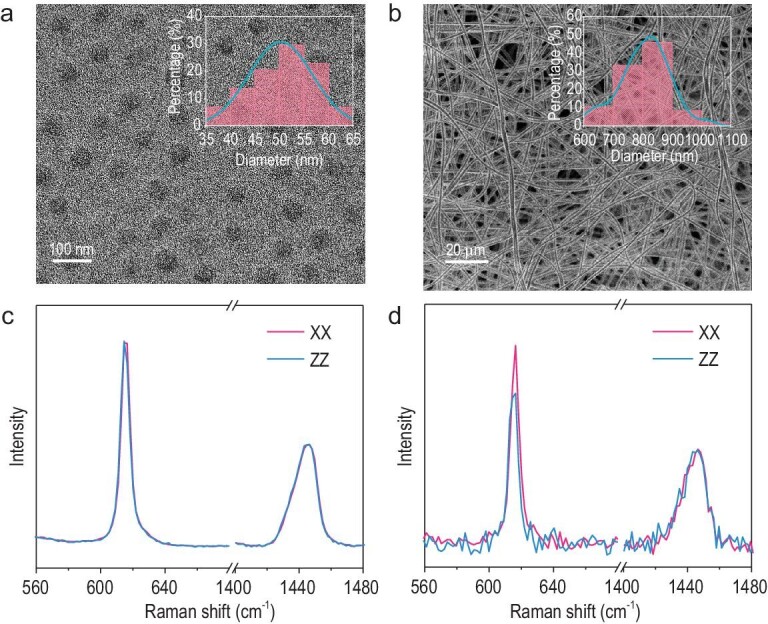
(a) TEM image of the spherical PS/PEO-*b*-PS/resol core@corona micelles. (b) SEM image of the oriented PS/PEO-*b*-PS/resol polymer nanofibers film. Polarized Raman spectra of (c) non-ordered PS film and (d) electrospun PS nanofiber (the accuracy of the measurements was confirmed by the equivalent intensity of the XX and ZZ spectrum at the band of 1450 cm^−1^ for both samples).

The synthesis of ordered mesoporous nanofibers is based on a bottom-up self-assembly strategy (Fig. 1b). The PS (Mw = ∼192 000 g mol^−1^) was dispersed in a mixed polar solvent of tetrahydrofuran (THF) and dimethyl formamide (DMF). The diblock copolymer PEO-*b*-PS (Mw = 29 960 g mol^−1^, Figs S1 and S2) and resol (Mw <500 g mol^−1^) were then added. The dispersed PS served as nucleation sites, at which the hydrophobic PS block of PEO-*b*-PS was incorporated. Meanwhile, the hydrophilic PEO part pointed outward, and attracted resol molecules with hydrogen bonding. Consequently, a nanoscale core@corona (PS/PEO-*b*-PS/resol, PS/PEO-*b*-PS is the core and resol is the corona) micelle structure with a uniform size of ∼50 nm was formed (Fig. 2a). In typical approaches for constructing mesoporous structures via EISA [36,37], the micelle dispersion was then subjected to solvent evaporation, triggering the close-packing assembly of micelles. Mesoporous carbon with spherical pores (diameter ∼37 nm) can be obtained by removing the soft-template (i.e. PS/PEO-*b*-PS) via pyrolysis (Fig. S3). However, limited by the stacking effect during the solvent evaporation process, it is difficult to change pore size without changing micelle composition, and regulate the pore orientation. The obtained carbon products are usually in the particle form lacking long-range order at the electrode scale. Here, electrospinning was applied. The viscous micelle dispersion was electrically charged under high-voltage (20 kV), and ejected from the spinneret to the collecting roller. The rapid extensional flow of the micelle solution imposed strong hydrodynamic forces [38–40], which led to the longitudinal stretching and transversal contraction of the electrospun network. Solvent evaporation drove the contact and self-assembly of core@corona micelles, during jet traveling. The PS chains in different cores aligned with each other. The stretching caused disentanglement and axial orientation of the PS chains [41]. The aligned PS chains further worked as the skeleton and elongated the spherical PS/PEO-*b*-PS/resol micelles into nanowires (Fig. 1b). Solidification of the nanofibers also occurred as the jet traveled. Fast evaporation of volatile solvents and solidification process avoid orientation relaxation of PS chains. As a result, continuous nanofibers consisting of well-ordered axially-stretched micelles can be obtained. The resol works as the carbon source for the final carbon nanofiber. The as-spun oriented PS/PEO-*b*-PS/resol polymer nanofibers have a uniform diameter of ∼830 nm (Fig. 2b). The collected nanofibers can form a free-standing film with a size reaching 8 × 24 cm^2^ (Fig. S4). To confirm the stretching-induced molecular orientation of PS chains, confocal Raman spectroscopy measurements were performed. The intensities of the parallel-polarized spectra (XX and ZZ) in the spectral range of 600–640 cm^−1^ (Fig. 2c) for randomly oriented PS film are similar. By comparison, the intensity of the ZZ spectrum is lower than that of the XX spectrum (Fig. 2d), validating the global axial chain orientation of the electrospun PS.

**Figure 3.**
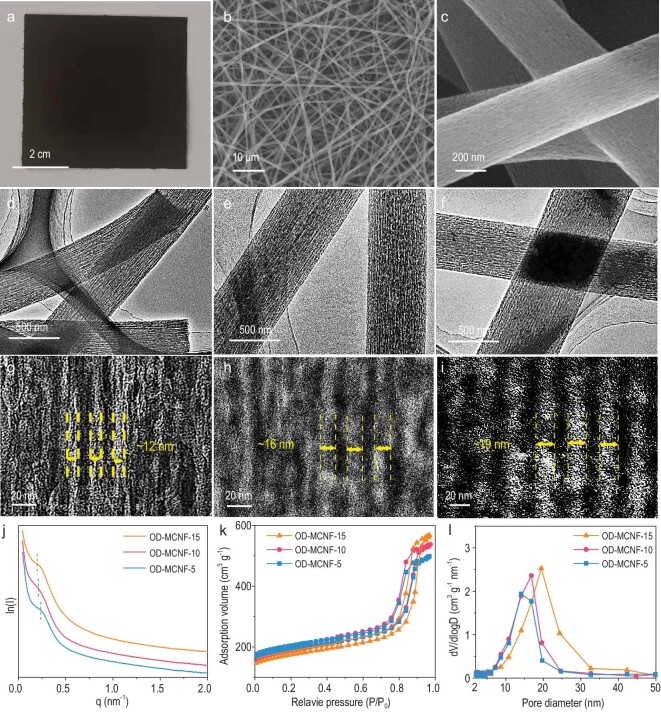
(a) Digital photo image of the flexible OD-MCNF-10 film. (b, c) SEM images of the OD-MCNF-10. TEM images of (d, g) OD-MCNF-5, (e, h) OD-MCNF-10 and (f, i) OD-MCNF-15. (j) SAXS patterns, (k) nitrogen-sorption isotherms and (l) pore size distributions of OD-MCNF-5, OD-MCNF-10 and OD-MCNF-15.

The presence of well-aligned micelles in oriented PS/PEO-*b*-PS/resol polymer nanofibers was further verified by the porous structure of the carbon product. The soft-template (i.e. PS/PEO-*b*-PS) can be removed by high-temperature pyrolysis of oriented PS/PEO-*b*-PS/resol polymer nanofibers, leaving behind mesopores. Meanwhile, resol molecules between micelles underwent crosslinking and carbonization, resulting in the formation of a carbon scaffold. Ordered mesoporous carbon nanofibers (OD-MCNF-x, x represents the weight % of PS homopolymer relative to PEO-*b*-PS) were obtained. The carbon product retained the nanofiber morphology of the oriented PS/PEO-*b*-PS/resol polymer nanofibers, and the size of the free-standing carbon nanofiber film was limited to the pyrolysis chamber size (Fig. 3a). Uniform longitudinal mesopores were observed throughout every nanofiber as shown in the scanning electron microscopy (SEM) and transmission electron microscopy (TEM) images (Fig. 3b–f and Fig. S5). The diameter of the longitudinal mesopores increased from 12 to 19 nm when improving the relative mass ratio of PS from 5 to 15 wt% (Fig. 3g–i).

The global structure of OD-MCNF-x was characterized by small-angle X-ray scattering (SAXS) (Fig. 3j). The OD-MCNF-5 shows one resolved scattering peak at a q value of ∼0.26, which confirms the regular ordering of mesopores. The peak shifts to a lower q value with the increase of PS concentration, which is indicative of the increased interpore spacing. The nitrogen adsorption and desorption curves of the OD-MCNF-5, 10, and 15 (Fig. 3k) show a type-IV isotherm, suggesting the formation of a mesoporous structure. The pore size distributions calculated by the Barrett-Joyner-Halenda (BJH) method indicate that the uniform mesopore size increases from 15.1 to 20.4 nm as x increases from 5 to 15 (Fig. 3l and Table S1), which is in perfect agreement with the TEM observation. The X-ray diffraction (XRD) pattern of the OD-MCNF (Fig. S6) shows two diffraction peaks of the 002 and 101 reflections, corresponding to those of graphite. High-resolution TEM (HRTEM) imaging shows that the carbon framework of OD-MCNF consists of graphitic structures with an interlayer spacing of 0.34 nm (Fig. S7). Energy dispersive spectroscopy (EDS) element mapping images show the uniform distribution of C and O elements throughout the whole nanofiber (Fig. S8).

The successful preparation of oriented PS/PEO-*b*-PS/resol polymer nanofibers relies on assembly and stretching of supramolecular micelles during electrospinning. In the absence of PS, the jet of low molecule weight PEO-*b*-PS and resol assemblies were broken into segments under strong elastic stretching, resulting in the formation of beads rather than continuous fibers. The corresponding carbon products were irregular worm-like mesoporous carbon spheres (Fig. 1c and Fig. S9). Without the employment of PEO-*b*-PS, the huge hydrophilic/hydrophobic difference between resol and PS homopolymers generated PS microemulsion, resulting in the heterogeneous macrophase separation with resol in the as-spun nanofiber. The corresponding carbon products were non-ordered mesoporous carbon nanofibers (NO-MCNF, Fig. 1d and Fig. S10).

To demonstrate that the strategy can be generalized to prepare ordered mesoporous nanofibers of different materials, resol was replaced with other metal precursors such as tetraethyl orthosilicate (TEOS), tetrabutyl titanate (TBOT) and tungsten chloride (WCl_6_). The hydrolytic polycondensation of the precursors with the assistance of an acid catalyst (i.e. HCl) generated metal oligomers. The oligomers interacted with the hydrophilic PEO block via hydrogen bonding, resulting in the formation of micelles similar to PS/PEO-PS/resol. The oligomers were further polymerized during electrospinning, and then crystallized with the formation of solid metal oxide walls upon pyrolysis. Meanwhile, ordered longitudinal mesopores formed after the removal of the PS/PEO-PS by pyrolysis. The TEM, STEM images and the corresponding EDS element mappings (Fig. S11) unambiguously show the contour of the single-oriented mesoporous structure of metal oxides. Of note, the parallel mesopores were connected via tiny pores. This is also confirmed by nitrogen sorption measurements (Fig. S12 and Table S1). Results of the pore size distribution indicate the presence of dominant longitudinal mesopores with uniform pore size, as well as co-existing tiny pores with the size of ∼2–5 nm. The formation of connecting tiny pores was possibly caused by the decomposition of the PEO blocks penetrating the metal oxide framework during pyrolysis [42]. Aggregation and sintering of metal oxide nanocrystalline was another possible reason for the tiny pores in TiO_2_ and WO_3_ [43]. Such pore configuration is similar to that in vascular bundles, in which aligned macro channels are connected by pores. We tested the mass transportation of water in the mesoporous SiO_2_ nanofiber film (Fig. S13). The water droplet spreads quickly once contacting the film, and the wetted region reached ∼4 times larger than that in commercial silica fiber film after 10 s. The accelerated water motion is due to unimpeded longitudinal mesopores as well as structure hierarchy.

Reactive heteroatom-containing gases (e.g. NH_3_ and H_2_S) from thermal decomposition of thiourea can etch the carbon framework and create connections between parallel mesopores. Meanwhile, N and S can be co-doped in OD-MCNF-10 (OD-MCNF (N, S)), which generates electrochemically active sites. The ordered mesopores were retained after thiourea treatment, with N and S homogeneously distributed throughout the nanofibers (Fig. S14). X-ray photoelectron spectroscopy (XPS) survey spectrum also detected the presence of N (4.1 at.%) and S (1.7 at.%) dopants (Fig. S15, Tables S2 and S3). The OD-MCNF (N, S) shows a higher relative intensity of the D band (1330 cm^−1^) to G band (1580 cm^−1^) (I_D_/I_G_ = 1.32) than that of the undoped OD-MCNF-10 (I_D_/I_G_ = 1.21), indicating the increased number of defects after thiourea treatment (Fig. S16) [44]. Nitrogen sorption isotherms of the OD-MCNF (N, S) show a curve with a combination of type-I and IV, which indicates the hierarchical micro/mesoporous structure (Fig. S17a). The micropore size distribution curve of the OD-MCNF (N, S) shows a peak centered at 1.0 nm, and the mesopore size is ∼20.1 nm (Fig. S17b). These micropores originated from the decomposition of the penetrated PEO blocks, as well as the carbon etching of thiourea treatment. The structure hierarchy together with uniform heteroatom doping makes it ideal for EECSD applications.

### Lithium metal storage based on ordered mesoporous carbon nanofiber

**Figure 4.**
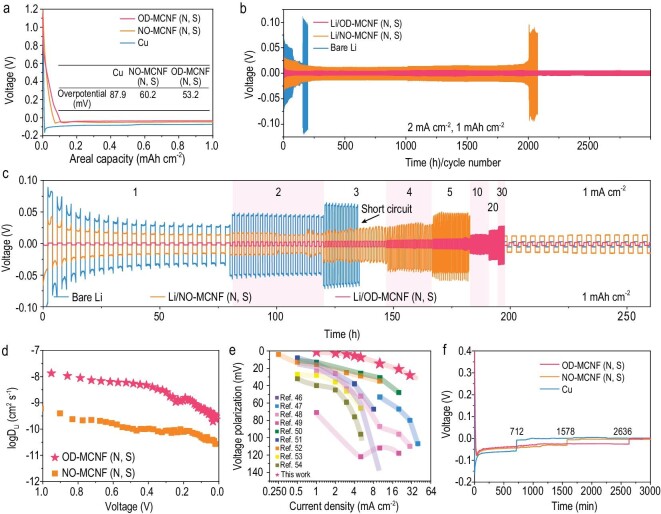
(a) Voltage profiles of Li plating on three different current collectors for the first cycle at a current density of 1 mA cm^−2^ with a capacity of 1 mAh cm^−2^ (The table in Fig. 5a shows the various nucleation overpotentials). Comparisons of: (b) cycling performance, (c) rate performance between bare Li, Li/OD-MCNF (N, S) and Li/NO-MCNF (N, S) symmetrical cells. (d) The Li-ion diffusion coefficient as a function of the state of discharging process. (e) Comparison of voltage polarization at various current densities between this work and recently reported work ([46]: CC-Zn-CMFs, [47]: LiCSMF, [48]: CCA, [49]: CNF-g-PSSLi, [50]: AgNWs/CNT, [51]: ZnO-MCNCF, [52]: mPPy-GO, [53]: N, S-CDs, [54]: PDDA-TFSI). (f) Electrochemical Li plating curves of the OD-MCNF (N, S), NO-MCNF (N, S) and Cu electrodes at 2 mA cm^−2^.

The OD-MCNF (N, S) was utilized as a host for lithium metal. The voltage profiles (Fig. 4a) of the Li plating process demonstrate the lowest nucleation overpotential (53.7 mV) of the OD-MCNF (N, S) compared to N and S co-doped NO-MCNF (NO-MCNF (N, S), Figs S18, S19, 58.9 mV) and Cu foil (160.6 mV). Moreover, the OD-MCNF (N, S) electrode also exhibited a largely reduced deposition overpotential (53.2 mV) and can withstand at least 200 cycles without obvious overpotential and capacity change (Fig. S20). By contrast, a much larger deposition hysteresis was observed for NO-MCNF (N, S) (60.2 mV) and Cu foil (87.9 mV), respectively. Such difference originated from the uniform Li plating in OD-MCNF (N, S), which can be confirmed by *ex-situ* SEM observation of morphological evolution across one plating/stripping cycle (Fig. S21). The initial surface of OD-MCNF (N, S) was relatively rough with partially exposed mesopores (Fig. S21b, c). When the deposition capacity reaches 1–2 mAh cm^−2^ (Fig. S21d–g), the surface became smooth due to the infiltration of Li into the porous framework [45]. A few Li nanodots began to appear on the surface of the OD-MCNF (N, S). With deposition capacity increasing (Fig. S21h, i), Li nanodots grew further. When the deposition capacity reached 10 mAh cm^−2^ (Fig. S21j, k), a smooth Li coating layer on the OD-MCNF (N, S) substrate formed, and tended to fill the gap between nanofibers, resulting in an even Li metal anode without dendrite formation. The lithium metal disappeared gradually during the following stripping process (Fig. S21l–o), and led to OD-MCNF (N, S) with a rough surface, evidencing excellent reversibility.

The cycling stability was further verified by a symmetric cell with OD-MCNF (N, S) wetted by molten lithium (Li/OD-MCNF (N, S)) as both anode and cathode (Fig. 4b and Fig. S22). The Li/OD-MCNF (N, S) symmetric cell exhibited a low overpotential (∼13 mV in the initial cycles), which stabilized at ∼5 mV for more than 3000 cycles (3000 h). In contrast, the Li/NO-MCNF (N, S) symmetric cell required a larger overpotential, and failed within less than 200 h. Moreover, OD-MCNF (N, S) also had outstanding reaction kinetics, showing ultra-low overpotentials of ∼15, ∼21 and ∼28 mV at rather high current densities of 10, 20 and 30 mAh cm^−2^, respectively (Fig. 4c and Fig. S23). Nevertheless, NO-MCNF (N, S) cells required fairly high overpotentials of ∼24, ∼31 and ∼48 mV to reach low current densities of 3, 4 and 5 mAh cm^−2^, respectively. Pure Li cells cannot even work at a current density higher than 3 mA cm^−2^. Such a difference suggests that ordered pores are essential to decrease diffusion impedance and deliver fast reaction kinetics. To achieve higher areal energy density, thickening the film can increase the active load per unit area. The OD-MCNF (N, S) host with double thickness was prepared. Even if the deposition overpotential increased to ∼187, ∼192 and ∼195 mV at 10, 20 and 30 mAh cm^−2^, the OD-MCNF (N, S) with double thickness can still work for a long cycle life (Fig. S24). However, the deposition overpotentials of NO-MCNF (N, S) with double thickness largely increased and the cell stopped working at 20 mAh cm^−2^, demonstrating the superiority of ordered mesoporous fibers. This is evidenced by the galvanostatic intermittent titration technique (GITT, Fig. 4d and Fig. S25) measurement. The calculated Li^+^ diffusion coefficient (D_Li+_) of OD-MCNF (N, S) is 1.34 × 10^−8^ cm^2^ s^−1^ at 1 V, which is ∼20 times higher than that of the disordered NO-MCNF (N, S) (6.17 × 10^−10^ cm^2^ s^−1^). To the best of our knowledge, the rate performance of this work is the best among those recently reported lithium metal composite anodes (Fig. 4e and Table S4) [46–54].

The electrochemical impedance spectroscopy (EIS) results suggested that the original Li/OD-MCNF (N, S) symmetric cell possesses a lower charge transfer resistance (R_ct_) of ∼17 Ω than that of the Li/NO-MCNF (N, S) (∼42 Ω) (Fig. S26a, c). Moreover, when doubling the electrode thickness, the R_ct_ of Li/OD-MCNF (N, S) with double thickness symmetric cell only increased to ∼87 Ω (Fig. S26e), while that of Li/NO-MCNF (N, S) with double thickness symmetric cell greatly jumped to ∼214 Ω (Fig. S26g). The chronoamperometry curves show that the Li^+^ transfer number (t_Li+_) of the OD-MCNF (N, S) reached 0.84 (Fig. S26b), which is larger than that of the NO-MCNF (N, S) (0.80, Fig. S26d). Li/OD-MCNF (N, S) with double thickness electrode still maintained a high Li^+^ transfer number of 0.80 (Fig. S26f). However, the t_Li+_ of Li/NO-MCNF (N, S) with double thickness electrode was significantly reduced to 0.61 (Fig. S26h).

The Li/OD-MCNF (N, S) exhibits a prolonged Sand's time of ∼2600 minutes, which is much longer than those of Li/OD-MCNF (N, S) and bare Cu electrodes (Fig. 4f), confirming the fast Li^+^ transport at the interface and evenly distributed local electric field. When the mesopores in Li/OD-MCNF (N, S) are full, the metal Li can smoothly grow on the surface of these nanofibers (Fig. S27). Considering that the OD-MCNF (N, S) and NO-MCNF (N, S) have similar composition and specific surface area, the above results indicate that the ordered mesoporous structure can largely promote Li^+^ transport, enhance interface wettability, and slow down dendrite growth because of unobstructed diffusion channels.

To further verify the feasibility of Li/OD-MCNF (N, S) in a practical battery system, Li/OD-MCNF (N, S)||LiFePO_4_ and Li/OD-MCNF (N, S)||LiNi_0.8_Co_0.1_Mn_0.1_O_2_ (NCM811) batteries were assembled and tested (Table S5). After activation at 0.2 C for 3 cycles, the Li/OD-MCNF (N, S)||LiFePO_4_ showed the highest capacity of 154 mAh g^−1^ and kept 100% of its capacity after 100 cycles at 1 C (Fig. S28a). However, the initial capacities of Li and Li/NO-MCNF (N, S) are 145 and 150 mAh g^−1^, respectively. Moreover, the Li/OD-MCNF (N, S)||LiFePO_4_ battery had the best rate performance, which can afford high capacity of 162, 159, 153, 136 and 119 mAh g^−1^ at the current density of 0.2, 0.5, 1, 2 and 5 C, respectively (Fig. S28b). On the contrary, the Li and Li/NO-MCNF (N, S) display obviously degraded rate performance. In addition, after activation at 0.2 C for 3 cycles, the Li/OD-MCNF (N, S)||NCM811 showed an original capacity of 190 mAh g^−1^ at 0.5 C and kept stable for 100 cycles with 95% capacity retention (Fig. S29a). However, only 82% and 87% of their initial capacities can be delivered for Li and Li/NO-MCNF (N, S) after 100 cycles. The Li/OD-MCNF (N, S)||NCM811 had the best rate performance (Fig. S29b). During cycling, the charge/discharge curves of Li/OD-MCNF (N, S)||NCM811 cell maintained well, however, the overpotential for Li/NO-MCNF (N, S)||NCM811 increases (Fig. S29c, d).

The Li/OD-MCNF (N, S)||NCM811 coin cells were dis-assembled and the Li/OD-MCNF (N, S) anode was analyzed (Figs S30–S32). XPS analysis confirms the formation of a LiF/Li_x_PO_y_F_z_ SEI layer. The formation of LiF/Li_x_PO_y_F_z_ in the SEI layer likely contributed to the enhanced cycling. XRD and SEM characterizations of the cycled Li/OD-MCNF (N, S) anode show the retention of the ordered mesoporous carbon structure.

Furthermore, a Li/OD-MCNF (N, S)||NMC811 pouch cell with designed capacity of 2.8 Ah was fabricated and tested under room temperature, as shown in Fig. S33 and Table S6. The initial energy density of this cell can reach 323 Wh kg^−1^ based on the whole cell weight. The cell can still deliver a high energy density of 284 Wh kg^−1^ after 120 cycles, corresponding to 88% capacity retention. The above results further demonstrate the advantage of ordered mesoporous structures as a lithium host.

### Analysis of lithium deposition mechanism

**Figure 5.**
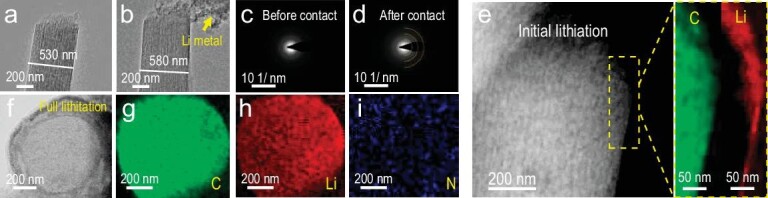
(a, b) *In-situ* TEM images and (c, d) corresponding SAED patterns of OD-MCNF (N, S) before and after contacting to Li/Li_2_O electrode. (e) STEM image and EELS mappings of OD-MCNF (N, S) at initial plating. (f–i) TEM image and EELS mappings of the cross-section of the Li plated OD-MCNF (N, S) obtained by FIB cutting.

**Figure 6.**
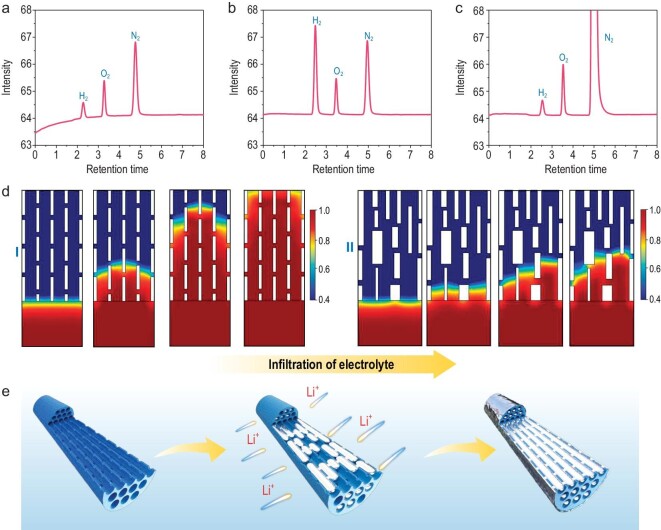
Chromatogram of gases with H_2_ after H_2_O titration on metallic Li of (a) Li/OD-MCNF (N, S) and (b) Li/NO-MCNF (N, S) in the Li/OD-MCNF (N, S)||NCM811 and Li/NO-MCNF (N, S)||NCM811 full coin cell after 100 cycles. (c) Gas chromatogram of certified H_2_ sample (200 ppm, Shanghai Haoqi Gas Co., Ltd). (d) The simulation results of the electrolyte transportation in cross-sectional view for the (Ⅰ) ordered mesoporous nanofiber and (Ⅱ) non-ordered mesoporous nanofiber using COMSOL Multiphysics. (e) Schematic illustration of the lithium plating process within OD-MCNF (N, S).

To further uncover the origin of the superior electrochemical properties of the OD-MCNF (N, S) host, *in-situ* TEM analysis of the Li plating process was carried out. A bias of −1 V was applied to initiate the plating process, once the OD-MCNF (N, S) contacted the Li/Li_2_O electrode (Fig. 5a, b). The diameter of OD-MCNF (N, S) increased from 530 to 580 nm. Diffraction rings were assigned to Li_2_O, which should originate from the oxidation of Li metal under the radiation of an electron beam as shown in the selected area electron diffraction (SAED) patterns (Fig. 5c, d). The STEM image and electron energy-loss spectroscopy (EELS) mappings of a single OD-MCNF (N, S) at initial lithiation shows that Li began to plate at the edge of the fiber (Fig. 5e). A focused ion beam (FIB) was applied to cut one OD-MCNF (N, S) with full lithiation to reveal the cross-section. The mesopore channels were filled and the Li signal was uniformly distributed in the interior of the nanofiber (Fig. 5f–i and Fig. S34). Excessive Li plating induced huge volume expansion and part of the OD-MCNF (N, S) fractured (Fig. S35). Li metal deposition involves two processes, the Li^+^ transports from the electrolyte to the surface of the current collector and then the electrochemical reduction of Li^+^. Based on previous reports, the channels are always the preferred nucleation sites for Li metal during extraction and deposition, and the pores are self-filled [45,55]. The prerequisite of uniform nucleation in pores is the unimpeded mass transfer of Li^+^. In the disordered porous system, dominant nucleation sites can be preferentially formed in the slow mass transfer region, resulting in excessive local growth of Li, and waste of pore space. The electronic conductive nanofiber network together with single-oriented ordered mesopores guarantees efficient electron and Li^+^ transport, which can decrease the polarization, slow down the accumulation of ‘dead’ Li and suppress the formation of Li dendrites. ‘Dead’ Li remaining in hosts of both Li/OD-MCNF (N, S)||NCM811 and Li/NO-MCNF (N, S)||NCM811 coin cells were quantified by the titration gas chromatography (TGC) method as reported previously (Fig. 6a–c) [56]. The amount of ‘dead’ Li in OD-MCNF (N, S) host was about 1.2 µg, while ‘dead’ Li in NO-MCNF (N, S) host was ∼5.8 times that of OD-MCNF (N, S) (6.9 µg). The EIS changes of the Li/OD-MCNF (N, S)||NCM811 were slight, confirming the acceptable impedance rise after cycling.

COMSOL multiphysics was further carried out to simulate the transfer of electrolyte in OD-MCNF (N, S) [57]. For OD-MCNF (N, S), the electrolyte can rapidly fill the whole parallel mesopores in either the planar or cross-sectional models. The mass transfer velocity of electrolyte in each parallel mesopore is basically identical because of the ordered mesoporous structure (Fig. 6d, Figs S36 and S37). However, for NO-MCNF (N, S), the mass transfer of electrolyte is quite nonuniform and some ‘pain spots’ of mass transfer emerge in both the planar and cross-sectional models. This hinders the infiltration of electrolyte, leading to much slower mass transfer velocity. The sluggish mass transportation further causes the generation of ‘dead’ Li in the ‘pain spots’, as confirmed in TGC results, which is harmful for the battery performance. The above results prove that the single-oriented ordered mesoporous nanofibers mimicking vascular bundles can greatly promote the mass transfer of electrolyte and provide abundant nucleation sites for uniform lithium deposition with little ‘dead’ volume (Fig. 6e).

## CONCLUSIONS

In conventional mesoporous material synthesis, a great number of experimental parameters have to be adjusted to find the appropriate hydrophobic/hydrophilic ratio of composited micelles to obtain the targeted mesoporous structure. In contrast, this newly designed electrospun strategy can directly manipulate the micelle structure and has great versatility to fabricate ordered mesoporous nanofibers with various compositions. This is the first time that an electric field has been introduced to adjust the micelle structure, representing a great breakthrough for the synthetic methodology of mesoporous materials. The ordered structure over centimeter-scale together with 3D conductive network enables uniform lithium nucleation and expedite Li^+^ transport. The corresponding lithium symmetric cell exhibits excellent cycling stability over 3000 h (3000 cycles) and ultra-low overpotentials of ∼15, ∼21 and ∼28 mV even at high current densities of 10, 20 and 30 mAh cm^−2^, which performs better than recently reported cells. The D_Li+_ of OD-MCNF (N, S) is one order of magnitude larger than that of the disordered counterpart. Our work demonstrates the power of nanoscale biomimicry of vascular structure for advanced EECSDs and beyond.

## METHODS

### Synthesis of ordered mesoporous carbon nanofibers (OD-MCNF)

In a typical synthesis, 1.0 g of PEO_117_-*b*-PS_240_ (Mw = 29 960 g mol^−1^, polydispersity index = 1.14; for ^1^H nuclear magnetic resonance spectra and gel permeation chromatography trace (Figs S1, S2)) and 0.10 g of PS were added in a mixed solvent containing 0.8 g of DMF and 1.2 g of THF under stirring. Then 2.5 g of resol solution was added with stirring for 10 min to form a homogeneous solution. During electrospinning, the high voltage, feeding rate and distance between the collector and the stainless-steel needle were set at 20 kV, 0.6 mL h^−1^ and 12 cm, respectively. Then, the as-made polymer membranes were thermally treated at 70 and 100°C successively to solidify the framework. Subsequently, the fiber membrane was annealed at 350°C for 2 h and then 800°C for 2 h with a ramp of 1°C min^−1^ in a vacuum environment, giving rise to the final product OD-MCNF.

### Synthesis of N and S co-doped OD-MCNF (OD-MCNF (N, S))

Co-doping of N and S elements in the OD-MCNF was achieved via pyrolysis with thiourea. In a typical synthesis, a mixture containing 0.1 g of OD-MCNF films and 1.0 g of thiourea was annealed at 800°C for 3 h with a ramp of 10°C min^−1^ under N_2_ atmosphere, giving rise to the OD-MCNF (N, S).

## Supplementary Material

nwae081_Supplemental_File
