# Supplementary material for: Ordered mesoporous nanofibers mimicking vascular bundles for lithium metal batteries
Source: Natl Sci Rev. 2024 Mar 11;11(5):nwae081. doi: 10.1093/nsr/nwae081 (PMC10989666; doi:10.1093/nsr/nwae081)
Supplement: nwae081_Supplemental_File [file nwae081_supplemental_file.pdf]

Ordered mesoporous nanofibers mimicking vascular bundles for lithium metal batteries

Xiaohang Zhu[1,†], Mengmeng Liu[1,†], Fanxing Bu[2], Xin-Yang Yue[3], Xiang Fei[1], Yong-Ning Zhou[4], Anqi Ju[1], Jianping Yang[1], Pengpeng Qiu[1], Qi Xiao[1], Chao Lin[1], Wan Jiang[1], Lianjun Wang[1], Xiaopeng Li[1,*] and Wei Luo[1,*]

[1]State Key Laboratory for Modification of Chemical Fibers and Polymer Materials, Institute of Functional Materials, College of Materials Science and Engineering, Donghua University, Shanghai 201620, China;
[2]Institute for Conservation of Cultural Heritage, Shanghai University, Shanghai 200444, China;
[3]Frontiers Science Center for Transformative Molecules, School of Chemistry and Chemical Engineering, Shanghai Jiao Tong University, Shanghai 200240, China;
[4]Department of Materials Science, Fudan University, Shanghai 200433, China

[*]**Corresponding authors.** E-mails: xiaopeng.li@dhu.edu.cn; wluo@dhu.edu.cn
[†]Equally contributed to this work.

Materials

Monomethyl poly(ethylene oxide) (Mw = 5,000 g mol^{-1}) (abbreviated as PEO-5000), polystyrene (Mw = 192,000 g mol^{-1}) and 2-bromo-2-methylpropionyl bromide were purchased from Aldrich. N,N,N’,N”,N”-Pentamethyl diethylenetriamine (PMDETA) was purchased from Acros Corp. Phenol, formalin solution (37.0-40.0 wt%), sodium hydroxide, petroleum ether (60-90 °C), Al_2O_3, ether, tetrahydrofuran (THF), hydrogen chloride (HCl), copper (I) bromide, pyridine, styrene, and dimethylformamide (DMF) were purchased from Sino-Pharm Chemical Reagent Co. Ltd. Styrene was further purified by filtrating through Al_2O_3 column. All other chemicals were used without further purification. Deionized water was used for all experiments.

Synthesis of resol precursor

The resol precursor with low-molecule-weight (Mw < 500 g mol^{-1}) was prepared according to a procedure reported previously [1]. In typical preparation, 0.34 g of 20% NaOH aqueous was added slowly into a round-bottom flask containing 1.60 g of phenol under vigorously stirring. Then, 0.34 g of 37 wt% formalin was dropped into the above solution. After being stirred at 70 °C for 1 h, the solution was cooled to room temperature and the pH value was adjusted to ~7.0 by HCl solution (1.0 M). H_2O in the solution was vacuum evaporated at 50 °C. The final product was dissolved in THF (60 wt%) for further using.

Synthesis of diblock copolymer poly(ethylene oxide)-*b*-polystyrene (PEO-*b*-PS)

The diblock copolymer was prepared by a reported atom transfer radical polymerization method [2]. The synthesis process of PEO-Br was performed as follows. First, 20.0 g of monomethoxy PEO-5000 and 20.0 mL of pyridine were dissolved in 120 mL of THF with stirring at 40 °C to form a clear solution. Then, the solution was placed in an ice-water bath and cooled to 0 °C. 6.0 g of 2-bromoisobutyryl bromide was added slowly under stirring within half an hour. Subsequently,

the solution was further stirred for 24 h at 30 °C. The solution was treated by vacuum evaporation to remove excess water and cold ether was added to precipitate the product, PEO-Br. The obtained PEO-Br was washed with ether and further dried in vacuum overnight. In the next step, 1.0 g of N,N,N',N",N"-Pentamethyl diethylenetriamine, 10.0 g of PEO-Br and 70 mL of styrene were added to an ampoules bottle, which was bubbled with N_2 for 30 min. After that, 0.20 g of CuBr was added into the solution quickly. The ampoules bottle was immersed in oil bath (110 °C) under stirring for 1.5 h. Then, the reaction solution was diluted by 100 mL of THF and filtered through Al_2O_3 column. An appropriate amount of petroleum ether was poured into the solution to precipitate the final product, PEO-*b*-PS block copolymer. The PEO-*b*-PS block copolymer was then dried in vacuum overnight.

Characterization

GPC investigation was carried on a BI-MWA type chromatographer with a refractive index detector using THF as an eluent. ^{1}H NMR spectra were taken on a DMX 500 MHz spectrometer (Bruker, Germany) with tetramethylsilane as an internal standard and $CDCl_3$ as a solvent. Small angle X-ray scattering (SAXS) measurements were recorded on a Nanostar U small-angle X-ray scattering system (Bruker, Germany) using Cu Kα radiation (40 kV, 35 mA). X-ray diffraction (XRD) patterns were obtained from a Bruker D2 diffractometer within 2θ ranging from 10 to 90°. Nitrogen sorption isotherms were measured at 77 K with a Micromeritics Tristar 3020 analyzer. Prior to the measurement, all the samples were degassed in a vacuum at 180 °C for 10 h. The Brunauer-Emmett-Teller (BET) method was utilized to calculate the specific surface areas. The non-local density functional theory (NLDFT) method was used to calculate the micropore size distributions and the Barrett-Joyner-Halenda (BJH) method was used to derive the mesopore size distribution. The total pore volumes were estimated from the adsorbed amount at a relative pressure P/P_0 of 0.992. Transmission electron microscopy (TEM) analysises were taken on a JEM-2100 F microscope (JEOL, Japan) operated at 200 kV. Scanning electron microscopy (SEM) images were taken on a TESCAN MAIA3 (Czech) microscope. Raman spectra were collected by using Raman microscopes (Renishaw, UK) with an excitation wavelength of 633 nm. X-ray photoelectron spectroscopy (XPS) was recorded on an AXIS ULTRA DLD XPS system and all calibrations were referenced to C1s line 284.8 eV.

Electrochemical Measurements

The assembly of Li metal symmetric coin cells

Li/OD-MCNF (N, S) and Li/NO-MCNF (N, S) composite anodes were prepared by infusing molten Li into the OD-MCNF (N, S) and NO-MCNF (N, S) hosts at 300 °C in an Ar-filled glove box, respectively. Electrochemical characterizations were done by using 2032-type coin cell. The obtained Li/OD-MCNF (N, S) and Li/NO-MCNF (N, S) were directly employed as free-standing composite anodes. The total weight of Li/OD-MCNF (N, S) or Li/NO-MCNF (N, S) anode was ~19 mg (~14 mg for Li and ~5 mg for OD-MCNF (N, S) or Li/NO-MCNF (N, S)). The weight of the bare Li electrode was around 46.5 mg. The Li/OD-MCNF (N, S), Li/NO-MCNF (N, S) and bare Li were assembled into a symmetric cell by two identical electrodes, respectively.

The test of Li metal symmetric coin cells

To evaluate the electrochemical deposition behavior, OD-MCNF (N, S), NO-MCNF (N, S), and

planar Cu foil were used as the working electrodes, and Li foil was used as the counter/reference electrode. The electrolyte contained 1 M lithium bis(trifluoromethanesulfonyl)imide (LiTFSI) in a mixture of 1,3-dioxolane (DOL) and dimethoxymethane (DME) (1:1 in volume) with the additive of 1 wt.% $LiNO_3$. The "Sand's time" of lithium dendrites nucleation uses the same method of electrochemical deposition. The galvanostatic intermittent titrations (GITT) were employed at a pulse of 0.1 mA for 10 min with 1 h interruption between each pulse. The Li^+ transference number was evaluated utilizing bare Li||Li, Li/OD-MCNF (N, S)||Li/OD-MCNF (N, S), and Li/OD-MCNF (N, S) with double thickness||Li/OD-MCNF (N, S) with double thickness symmetric cells combined by electrochemical impedance spectrum (EIS) before and after chronoamperometry (CA) test within the frequency of 0.01 to 1×10^5 Hz, and calculated by the following equation.

$$t_{\mathrm{Li+}} = I(\Delta V - I_0 R_0)/I_0(\Delta V - I_s R_s)$$

Where I_0 and R_0 are the initial current and resistance, respectively. ΔV is the voltage polarization applied (5 mV), I_s and R_s are the steady state current and resistance, respectively.

The assembly of Li metal full coin cells

For the full cells, the total weight of Li/OD-MCNF (N, S) or Li/NO-MCNF (N, S) anode was ~2.4 mg (~0.4 mg for Li and ~2 mg for OD-MCNF (N, S) or Li/NO-MCNF (N, S)) and a slurry contained commercial $LiNi_{0.8}Co_{0.1}Mn_{0.1}O_2$ (NCM811) or $LiFePO_4$ power, Super-p and PVDF binder with N-methyl-2-pyrrolidone as the solvent. Then, the well-mixed slurry was cast onto an Al foil to prepare the NCM811 or $LiFePO_4$ cathode. The active loading of cathodes is in Table S5. The electrolyte was composed of 1.0 M $LiPF_6$ in ethylene carbonate and diethyl carbonate (v:v = 1:1).

The assembly of Li metal full pouch cells

Similar to the Li/OD-MCNF (N, S)||NMC811 full coin cell, the Li/OD-MCNF (N, S)||NMC811 pouch cell was fabricated using ~7×6 cm^2 Li/OD-MCNF (N, S) film anode. The detailed parameters of the pouch cell are listed in the Table S6. The mass energy density of the pouch cell was calculated on the following equation:

$$E_g = \frac{VC}{\sum(m_i)}$$

E_g is the full-cell mass energy density. V is the output voltage, C is the capacity, m_i is the weight of each component of the pouch cell.

The measurement of inactive Li

Inactive Li was measured according to a titration gas chromatography (TGC) procedure reported previously[3]. For the TGC method, there are six main steps to quantify the inactive Li in Li/OD-MCNF (N, S) anode. (1) The Li/OD-MCNF (N, S)||NCM811 and Li/NO-MCNF (N, S)||NCM811 coin cells were first cycled at 0.2 C with the voltage ranging from 3.0 to 4.3 V for 99 cycles. During the 100th cycle, these coin cells were charged to 4.3 V and discharged to 0 V. (2) These coin cells were disassembled in an Ar-filled glovebox. The Li/OD-MCNF (N, S) and Li/NO-MCNF (N, S) anode were harvested and sealed in a 30 ml container with an inside pressure of 1 atm by a rubber septum which is stable against water, respectively. (3) After transferring the

sample container out of the glovebox, 0.5 ml of H_2O was injected into the container to react with the inactive Li completely. (4) A gas-tight syringe was used to transfer 100 μl of the resultant gas from the container into the gas chromatography system. (5) The amount of H_2 was measured by the gas chromatography. (6) The content of the metallic Li was determined by converting the corresponding H_2 amount according to certified H_2 (200 ppm, Shanghai Haoqi Gas Co., Ltd). All the processes minimize the potential damage and contamination during sample transfer, to obtain reliable results.

The content of metallic Li is quantified by the reaction: $2Li + 2H_2O = 2LiOH + H_2$ and two conditions are followed: (1) 1 p.p.m. = 4.08×10^{-8} mmol ml^{-1} (1 atm, 298 K); (2) container volume = 30 ml. The amount of inactive metallic Li in Li/OD-MCNF (N, S) and Li/NO-MCNF (N, S) are 1.2 and 6.9 μg, respectively.

Wettability simulation

The simulation was performed by COMSOL Multiphysics 5.3a software. In our simulation, the multiphase flow (electrolyte, 1 M LiTFSI in a mixture of DOL and DME (1:1 in volume) with 1 wt.% $LiNO_3$) through a porous medium (lithium metal host) and droplets on walls are main research objects where the porous structure and the wall strongly influence the wettability of the host to the electrolyte. This model uses a phase field method, to track the host/electrolyte interface and the diffusion of electrolyte in host. The capillary forces dominate over gravity throughout the simulation so that the interface moves upwards during the entire simulation. Besides, the wettability of the host was regulated by setting different contact angle at the channel walls. The contact angle for electrolyte/Cu and electrolyte/host is fixed at pi/2 rad and 3pi/8 rad, respectively. The physical models of the hosts were established by AutoCAD software, in which the size and distribution of the pores in host was considered. Before injecting the electrolyte, Ar atmosphere were filled in the host. The viscosity of the fluid applied in this simulation is 3×10^{-3} Pa·s. The applied pressure for simulation is 0 Pa.

Supplementary Figures

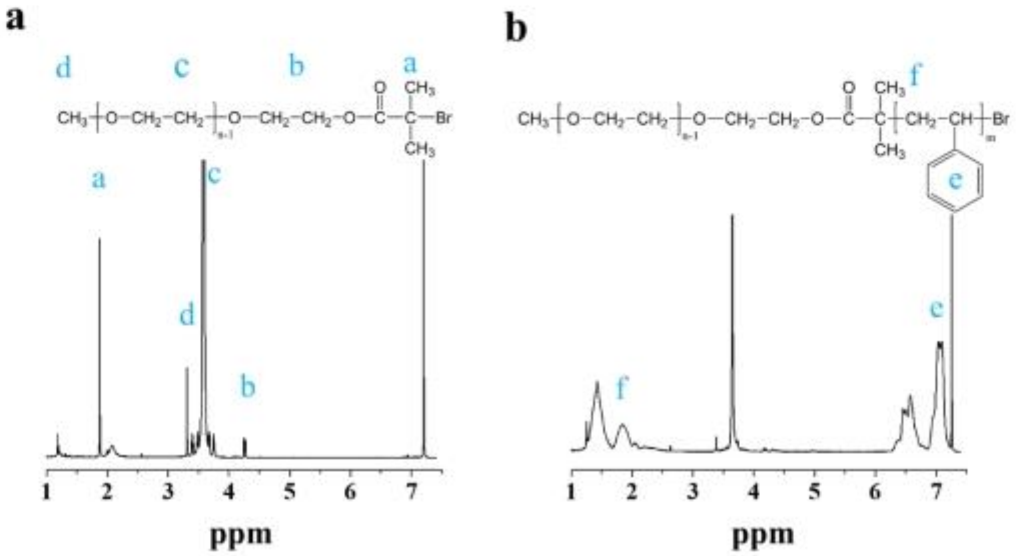

Fig. S1. ^{1}H NMR spectra of **a**, PEO-Br and **b**, the synthesized PEO_{117}-*b*-PS_{240}.

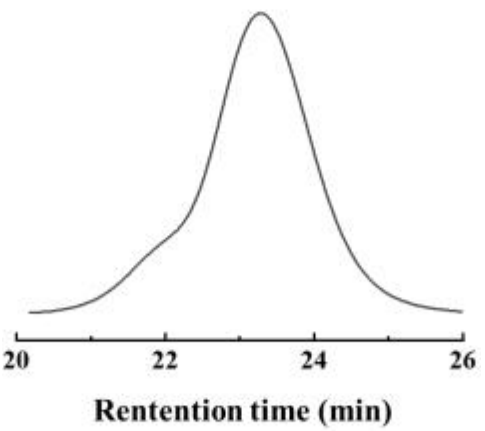

Fig. S2. The GPC trace of the synthesized PEO_{117}-*b*-PS_{240}.

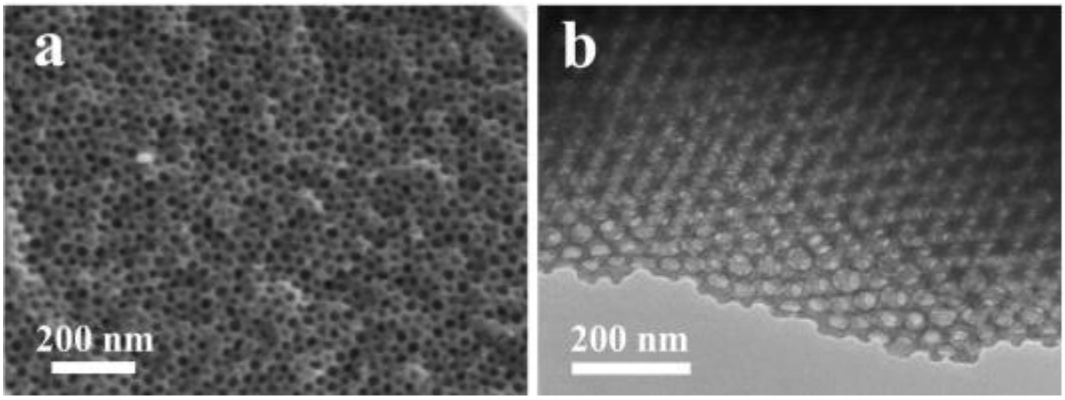

Fig. S3. **a**, SEM and **b**, TEM images of the mesoporous carbon powders prepared by evaporation induced self-assembly (EISA) method.

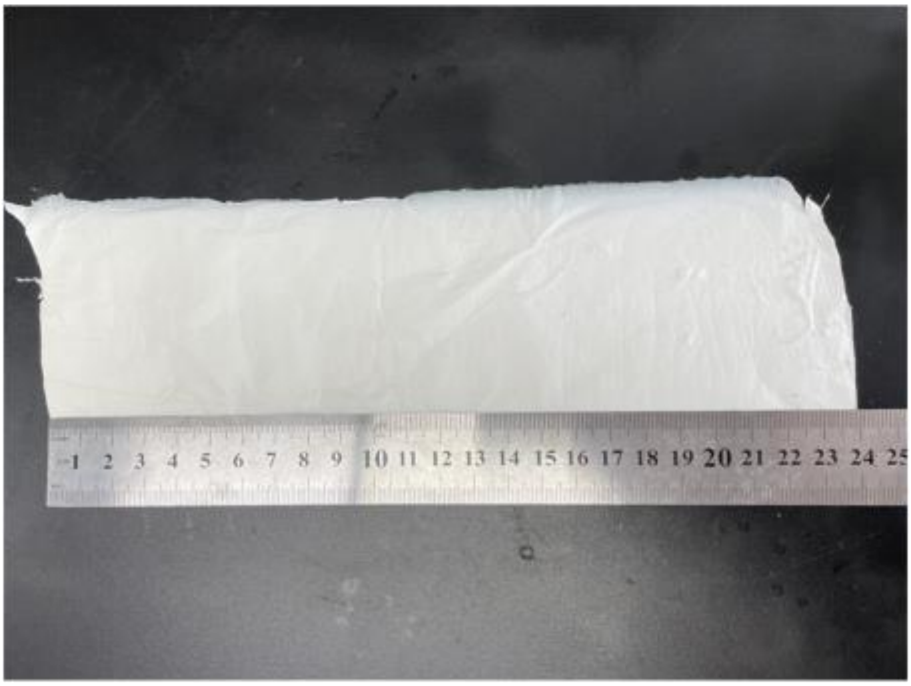

Fig. S4. Digital photo image of the as-spun oriented PS/PEO-*b*-PS/resol polymer nanofibers (OPNF) film.

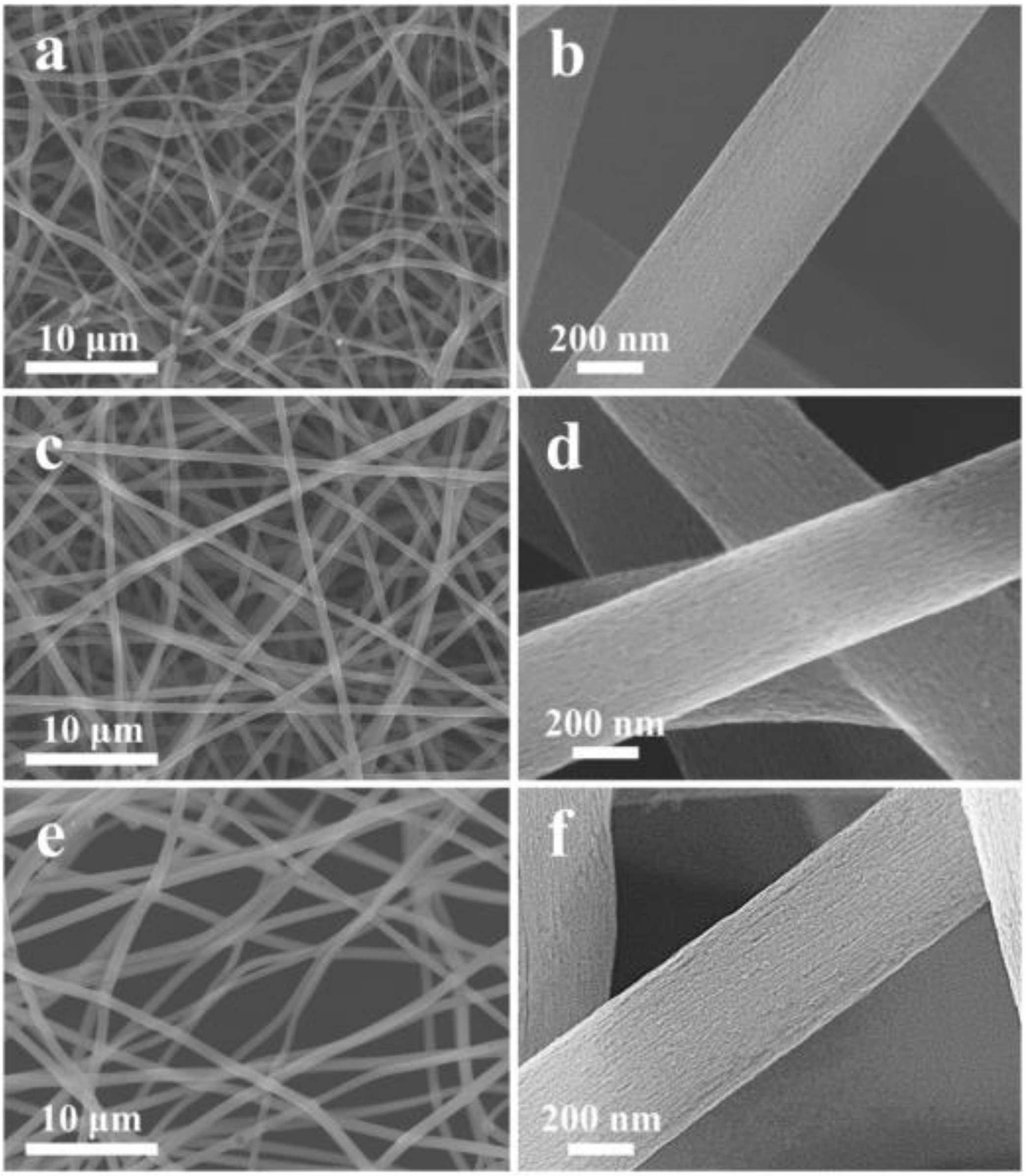

Fig. S5. The SEM images of **a, b**, OD-MCNF-5. **c, d**, OD-MCNF-10 and (e, f) OD-MCNF-15.

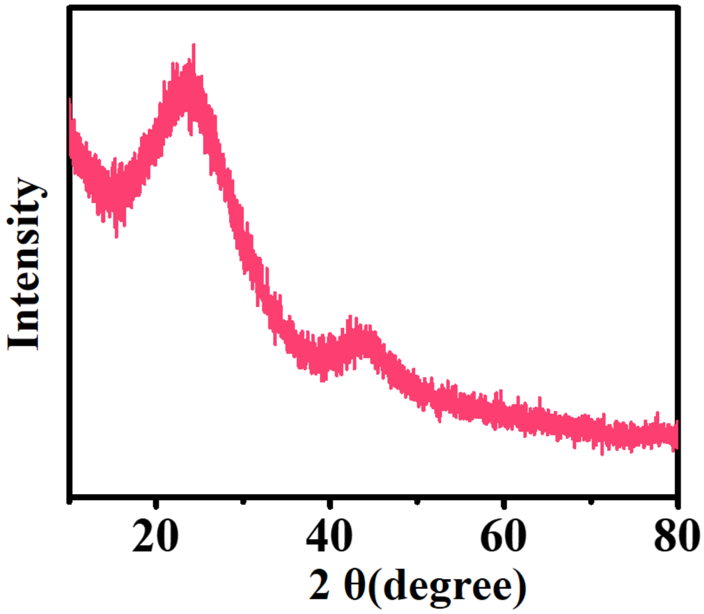

Fig. S6. XRD pattern of the OD-MCNF-10.

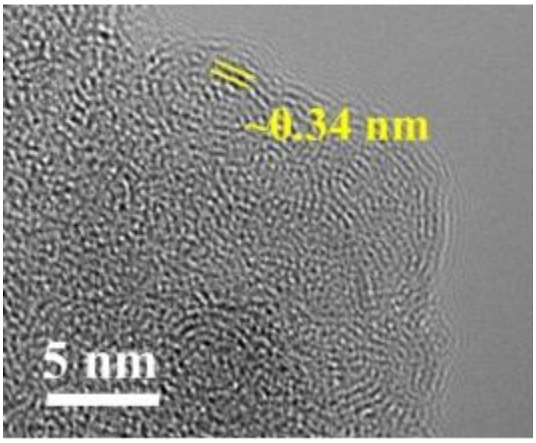

Fig. S7. High-resolution transmission electron microscopy (HRTEM) image of the OD-MCNF-10.

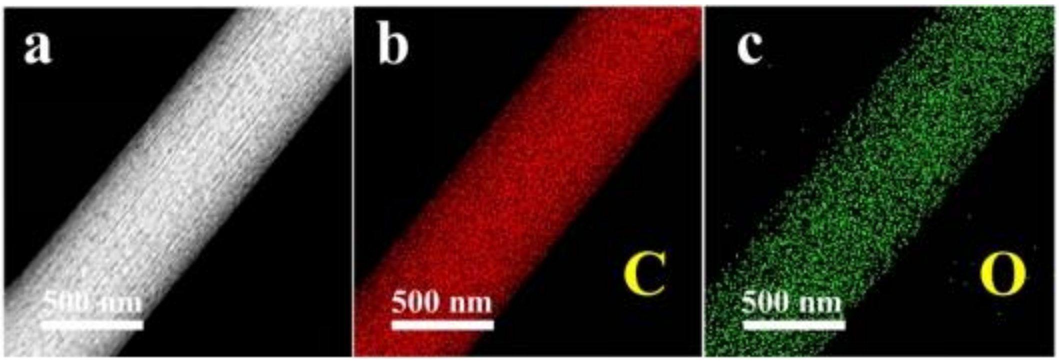

Fig. S8. **a**, Scanning TEM (STEM) and **b, c**, corresponding EDX mapping images of the OD-MCNF-10.

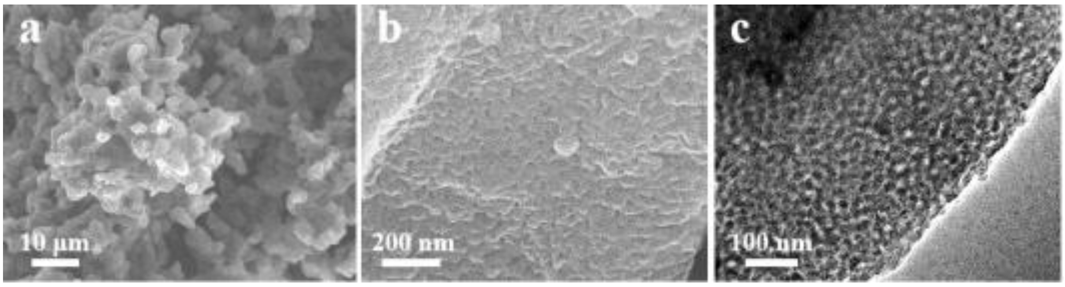

Fig. S9. a, b, SEM and **c**, TEM images of irregular worm-like mesoporous carbon spheres (WMCS).

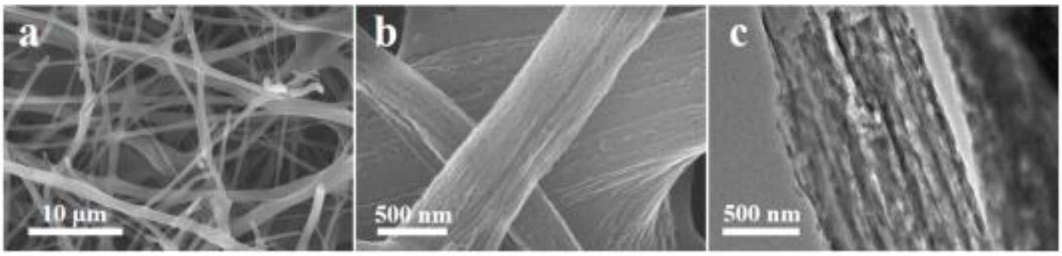

Fig. S10. **a, b**, SEM and **c**, TEM images of the non-ordered mesoporous carbon nanofibers (NO-MCNFs) with wide pore size distribution.

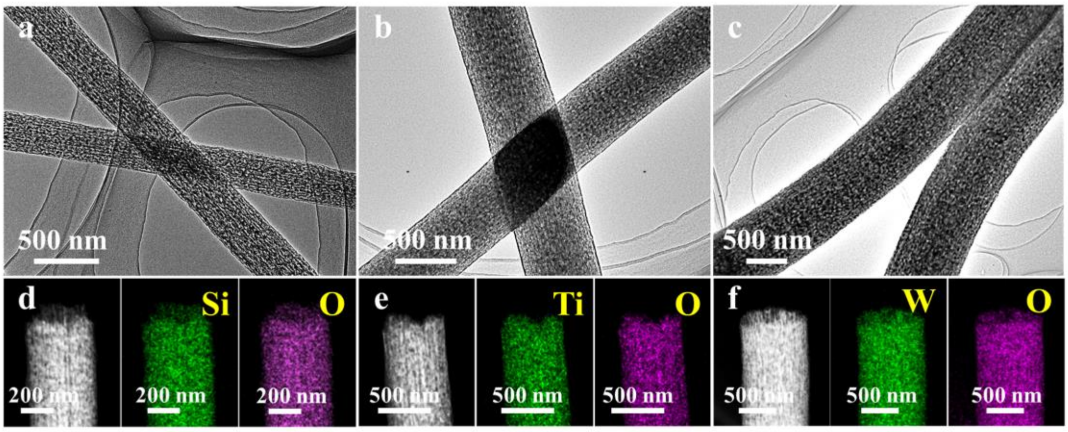

Fig. S11. TEM images and **d-f**, STEM images and the corresponding elemental mappings of ordered mesoporous SiO_2, TiO_2 and WO_3 nanofibers.

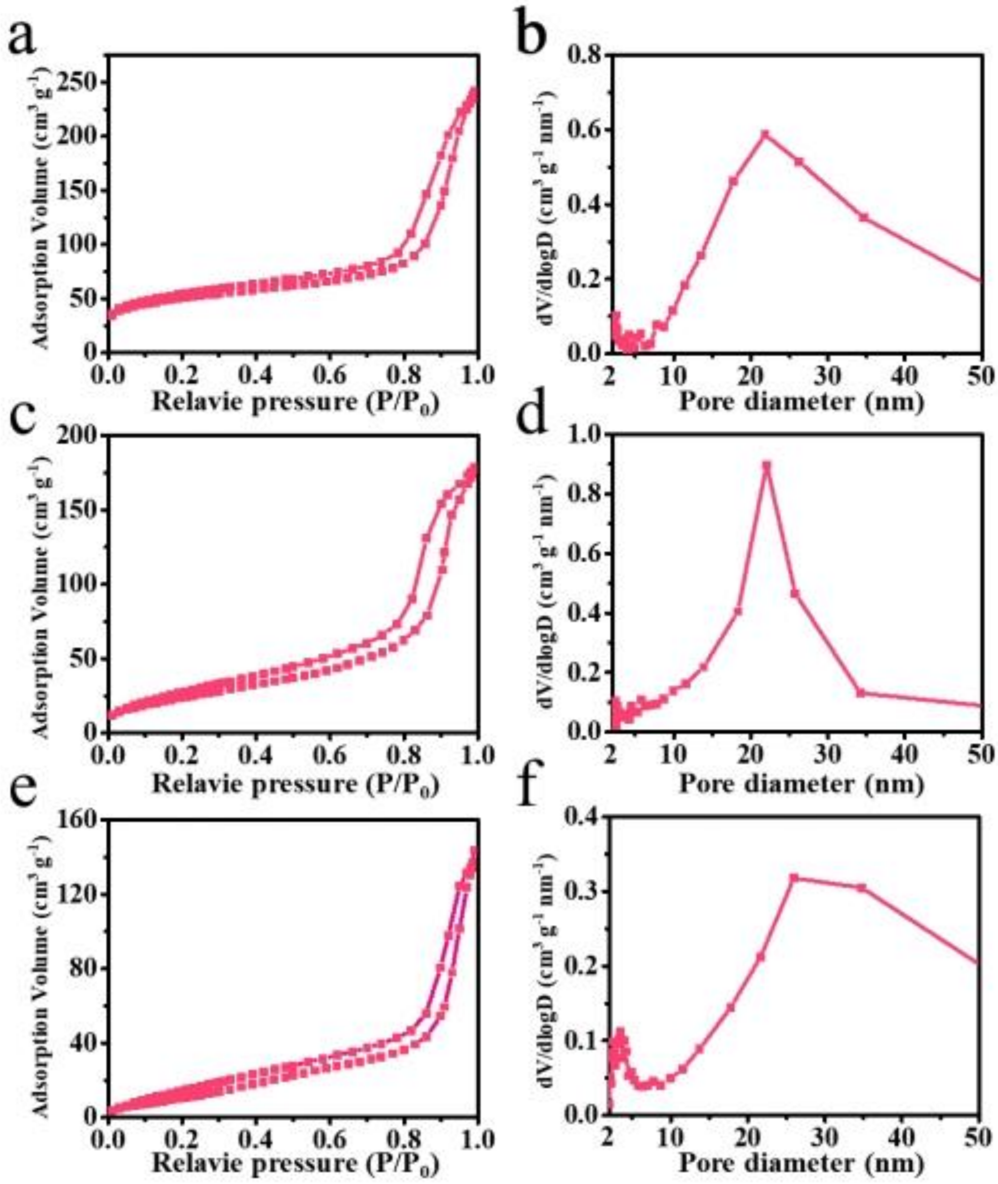

Fig. S12. a, c, e, Nitrogen adsorption-desorption isotherms and **b, d, f**, pore size distributions of order mesoporous SiO_2, TiO_2 and WO_3 nanofibers.

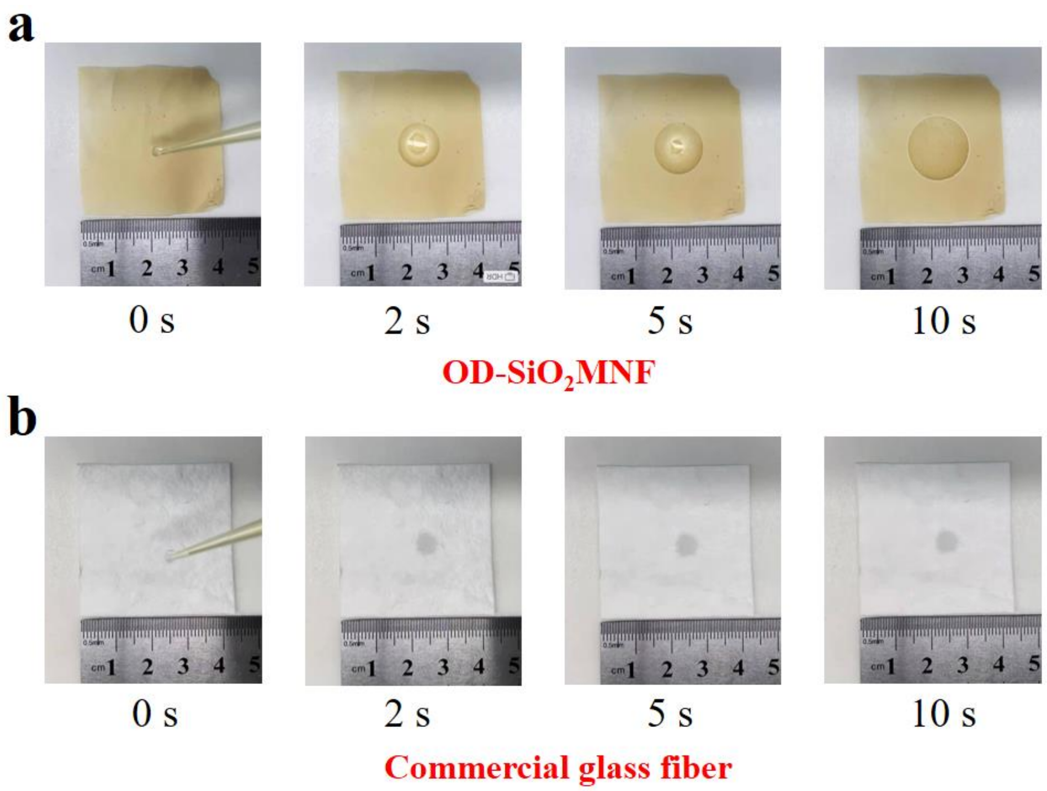

Fig. S13. Wetting behavior of water inside **a**, the ordered mesoporous SiO_2 nanofibers and **b**, commercial glass fiber.

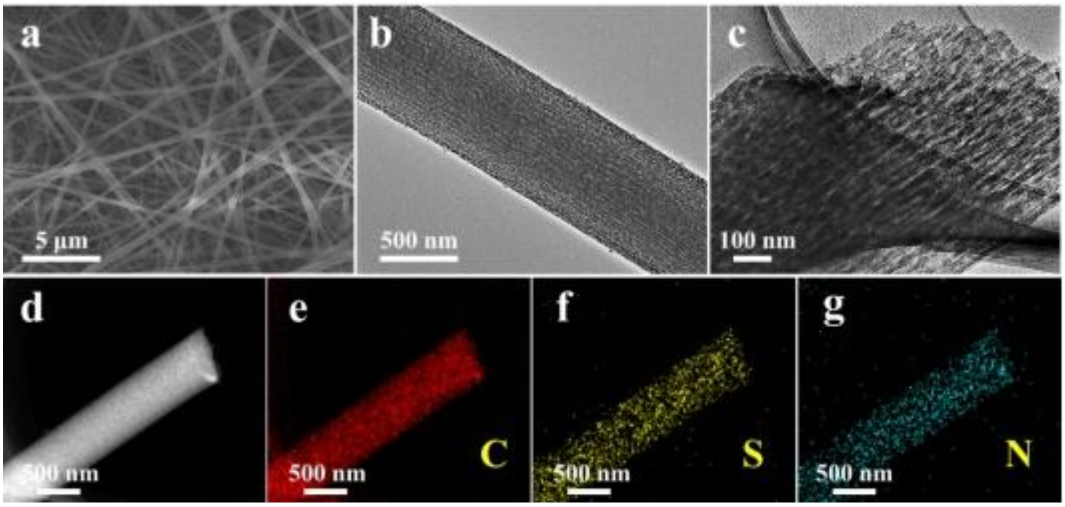

Fig. S14. **a**, SEM image, **b, c**, TEM images, **d**, STEM image and **e-g**, EDX element mappings of the OD-MCNF (N, S).

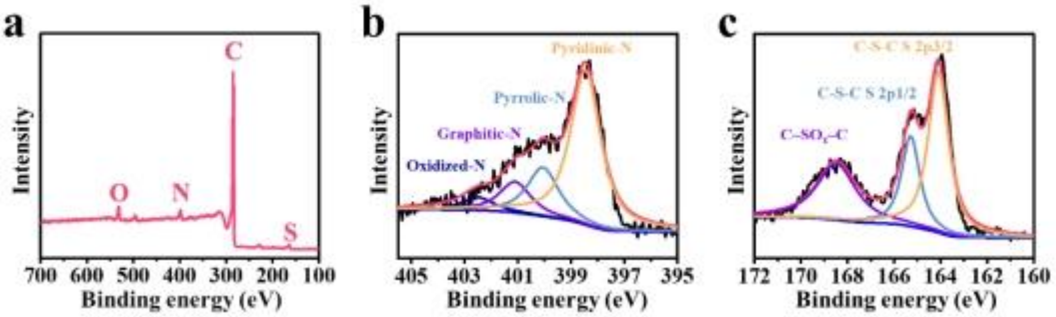

Fig. S15. **a**, XPS scan spectrum and the corresponding high-resolution **b**, N 1s and **c**, S 2p spectra of the OD-MCNF (N, S).

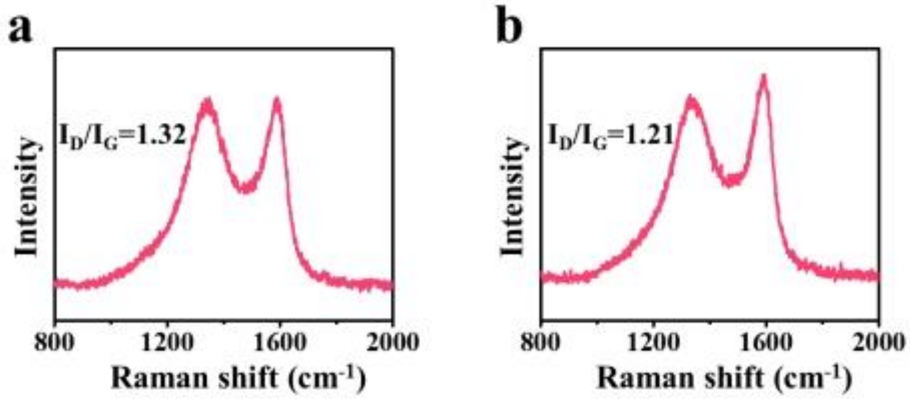

Fig. S16. Raman spectra of **a**, OD-MCNF (N, S) and **b**, OD-MCNF-10.

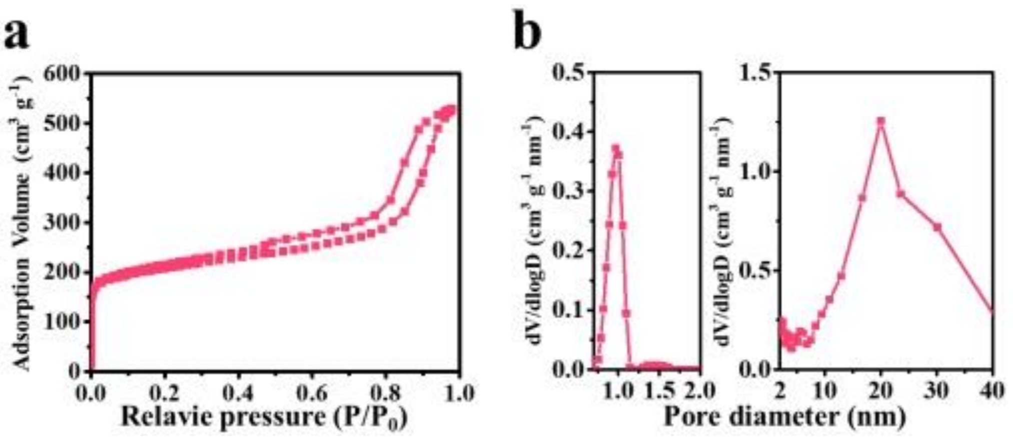

Figure S17. a, Nitrogen adsorption-desorption isotherm and **b**, micropore and mesopore size distributions of the OD-MCNF (N, S) calculated by NLDFT and BJH method, respectively.

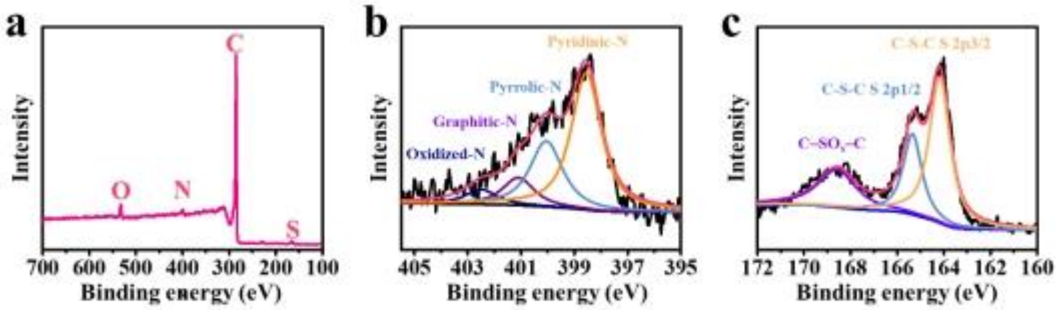

Fig. S18. **a**, XPS scan spectrum and the corresponding high-resolution **b**, N 1s and **c**, S 2p spectra of the NO-MCNF (N, S).

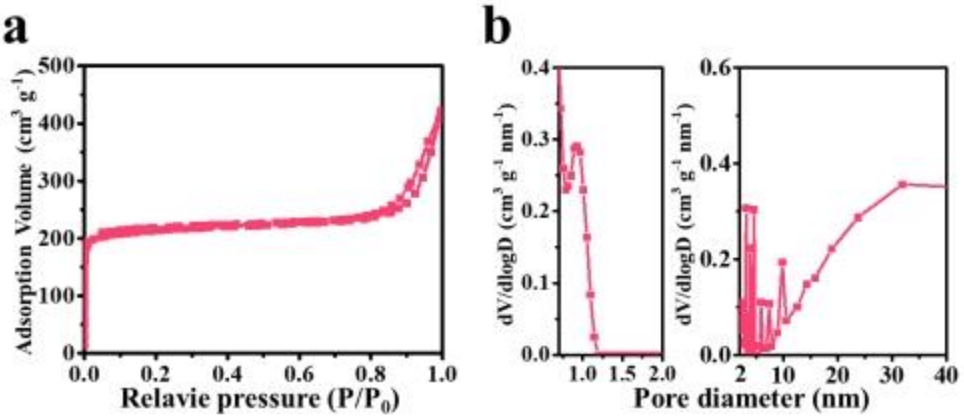

Fig. S19. **a**, Nitrogen adsorption-desorption isotherm and **b**, micropore and mesopore size distributions of the NO-MCNF (N, S) calculated by NLDFT and BJH method, respectively.

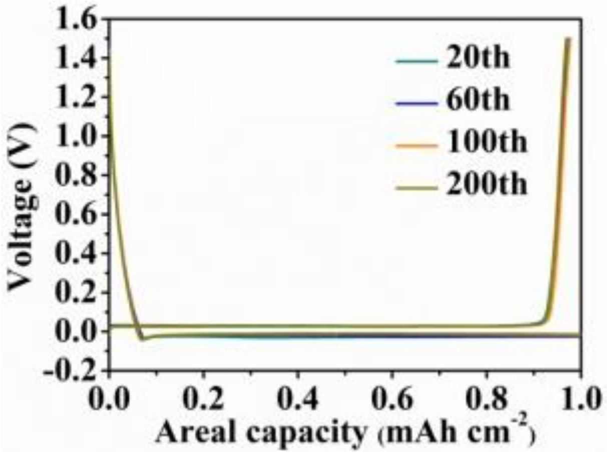

Figure S20. Selected electrochemical Li plating/stripping curves of the OD-MCNF (N, S) electrode at 1 mA cm^{-2} for a capacity of 1 mAh cm^{-2}.

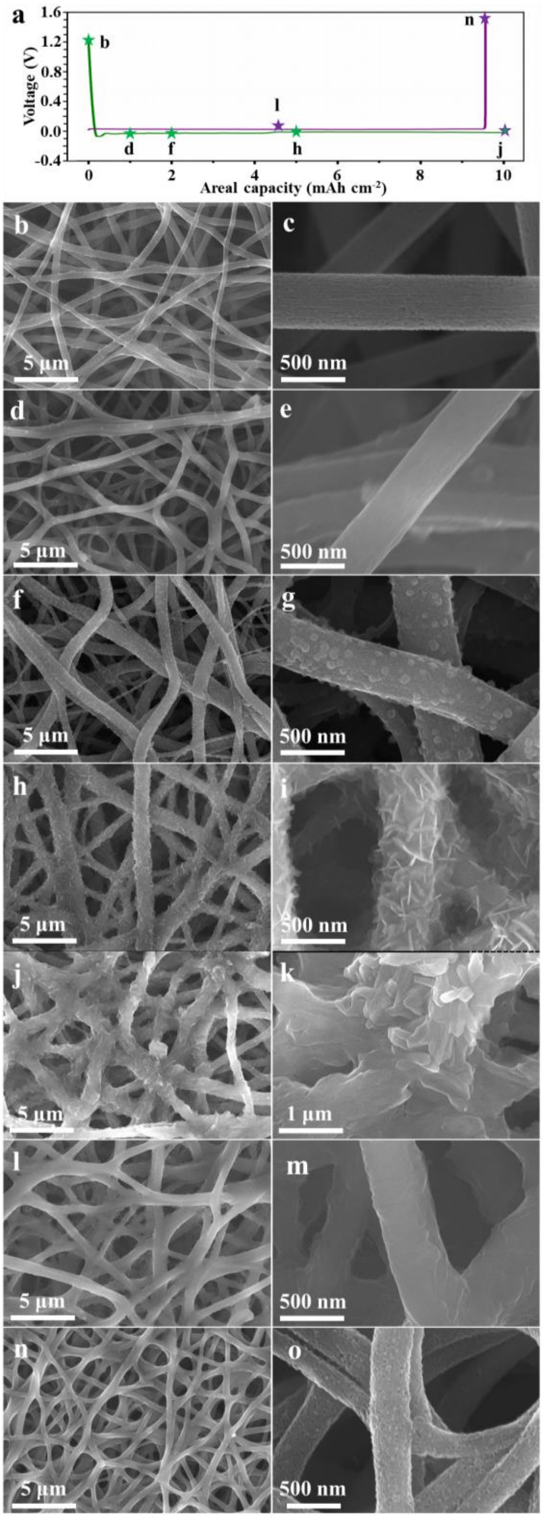

Fig. S21. **a**, Electrochemical Li plating/stripping curves of the OD-MCNF (N, S) electrode at 1 mA cm^{-2} for a capacity of 10 mAh cm^{-2}. (The states marked different letters in **a**, correspond to subsequent SEM images.) **b, c**, SEM images of the OD-MCNF (N, S) electrode. SEM images of the OD-MCNF (N, S) electrodes after being plated with **d, e**, 1 mAh cm^{-2}, **f, g**, 2 mAh cm^{-2}, **h, i**, 6 mAh cm^{-2}, and **j, k**, 10 mAh cm^{-2} of Li metal. SEM images of the anodes after stripping with (l, m) 5 mAh cm^{-2}, and

(n, o) 10 mAh cm^{-2} (i.e., recharged to 1.5 V) from the OD-MCNF (N, S) electrode with Li.

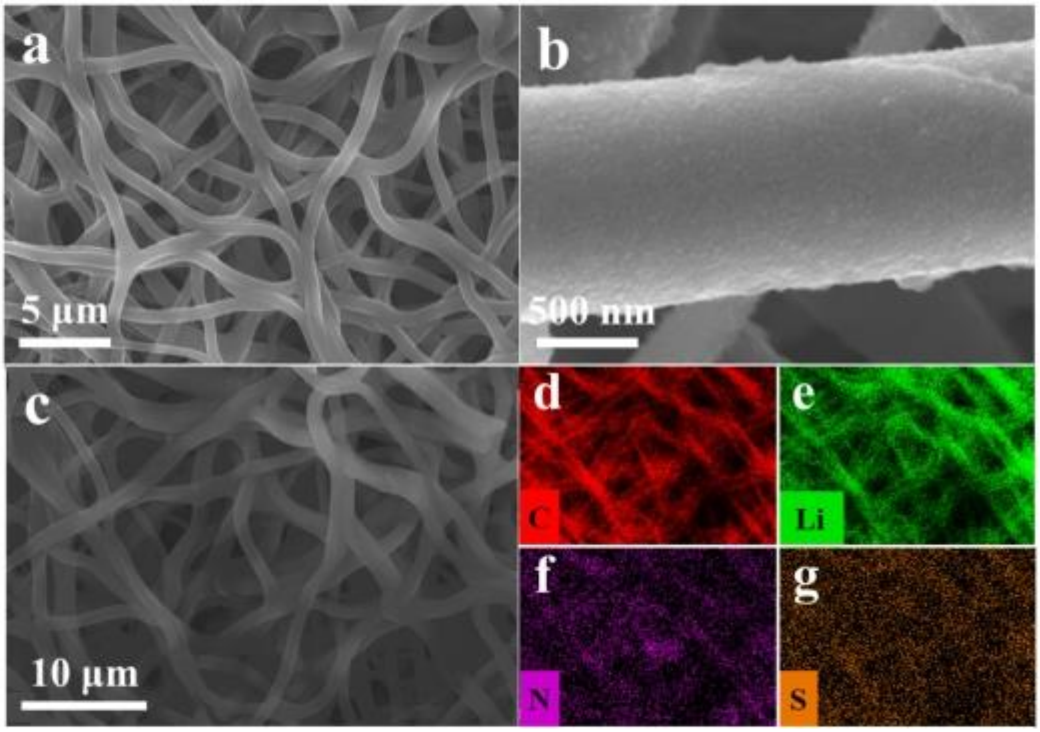

Fig. S22. a, b, SEM images, **c**, high-angle annular dark field SEM (HAADF-SEM) and **d-g**, corresponding elemental mappings of the Li/OD-MCNF (N, S).

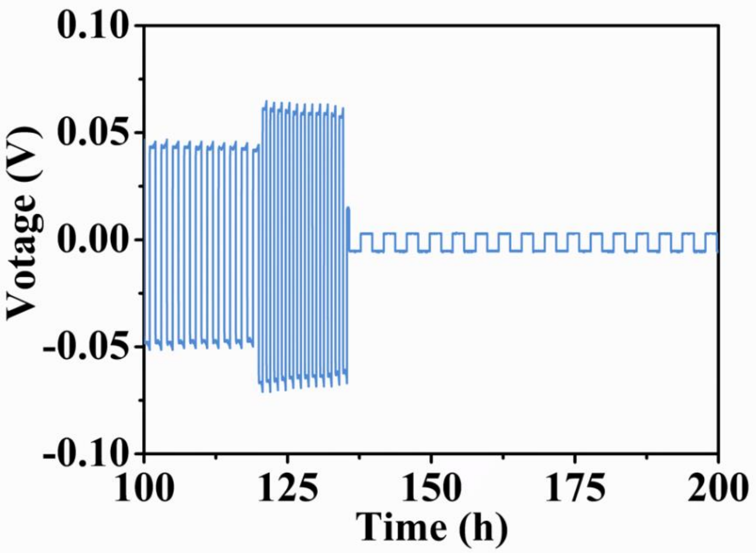

Fig. S23. Magnified rate performance of bare Li symmetrical cells.

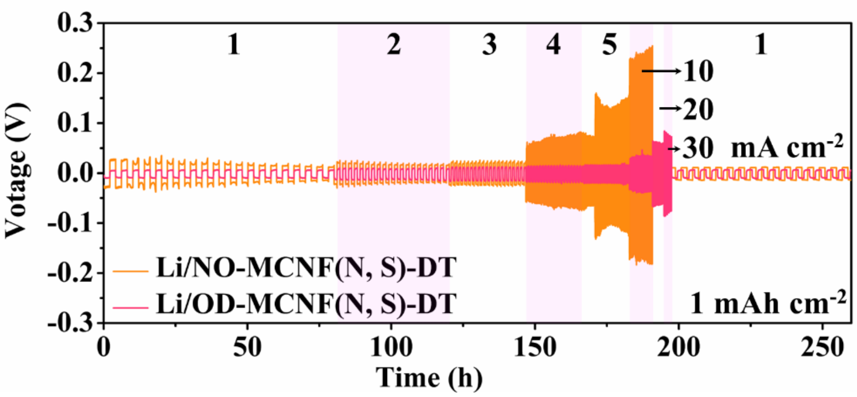

Fig. S24. Comparisons of rate performance between Li/OD-MCNF (N, S) with double thickness and Li/NO-MCNF (N, S) with double thickness symmetrical cells.

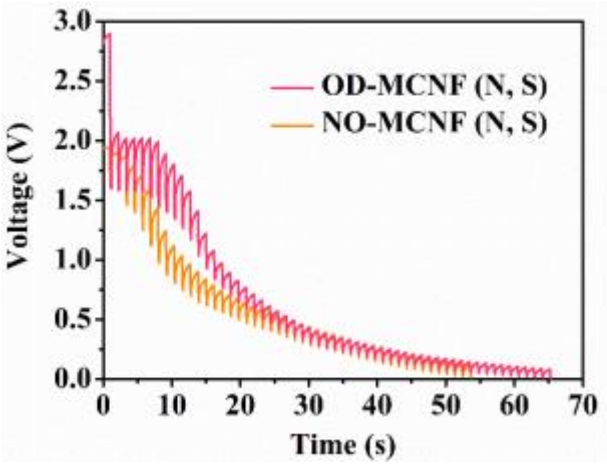

Fig. S25. GITT profiles of OD-MCNF (N, S) and NO-MCNF (N, S) during the discharging process.

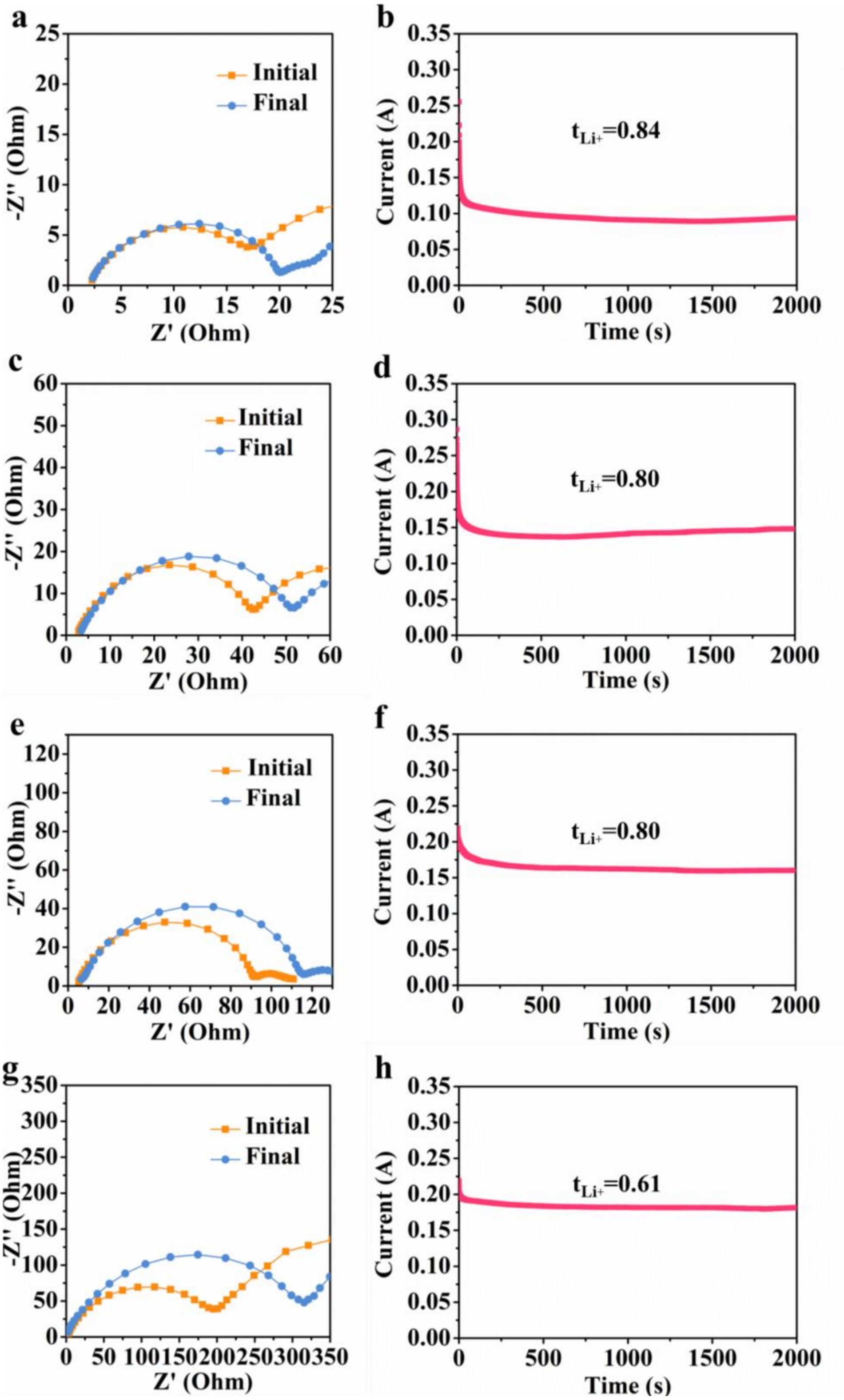

Fig. S26. The impedance results of: **a**, Li/OD-MCNF (N, S), **c**, Li/NO-MCNF (N, S), **e**, Li/OD-MCNF (N, S) with double thickness, and **g**, Li/NO-MCNF (N, S) with double thickness symmetric cells before and after chronoamperometry measurement. Chronoamperometry measurement results of: **b**, Li/OD-MCNF (N, S), **d**, Li/NO-MCNF (N, S), **f**, Li/OD-MCNF (N, S) with double thickness, and **h**, Li/NO-MCNF (N, S) with double thickness symmetric cells.

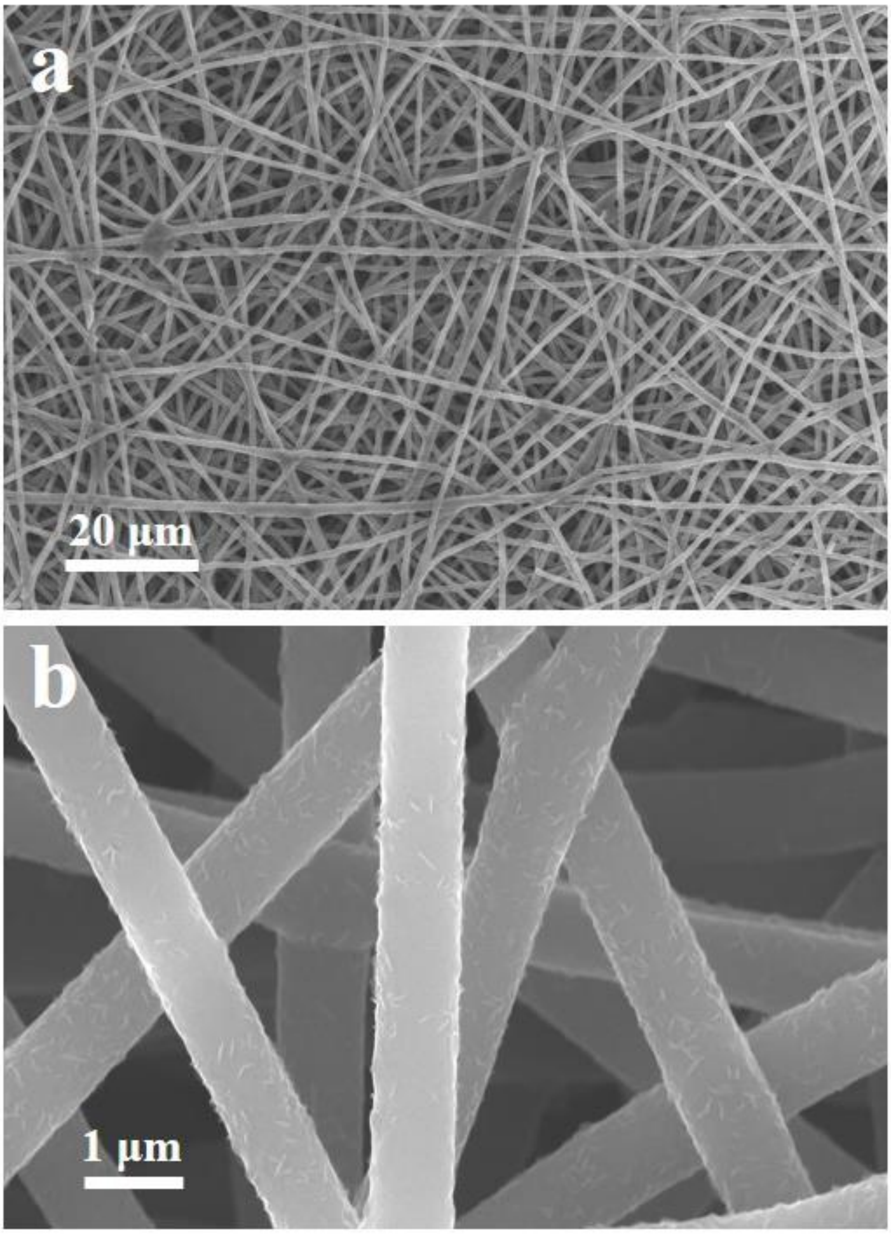

Fig. S27. SEM images of plated OD-MCNF (N, S) electrode. The electrode was first plated/stripped for 5 cycles and then plated at 2 mA cm^{-2} with 5 mAh cm^{-2}.

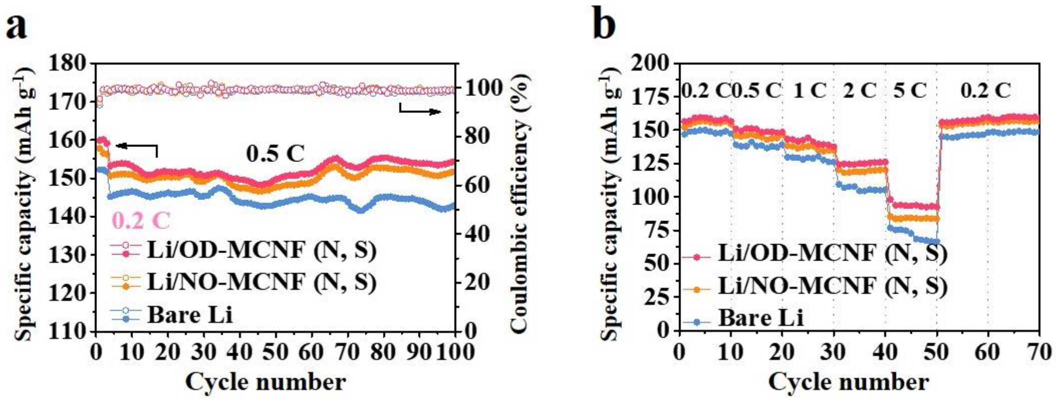

Fig. S28. **a**, Long-term cycling performances and **b**, rate performances of full coin cells constituted of various anodes with $LiFePO_4$ cathode with the voltage ranging from 2.0 to 4.0 V.

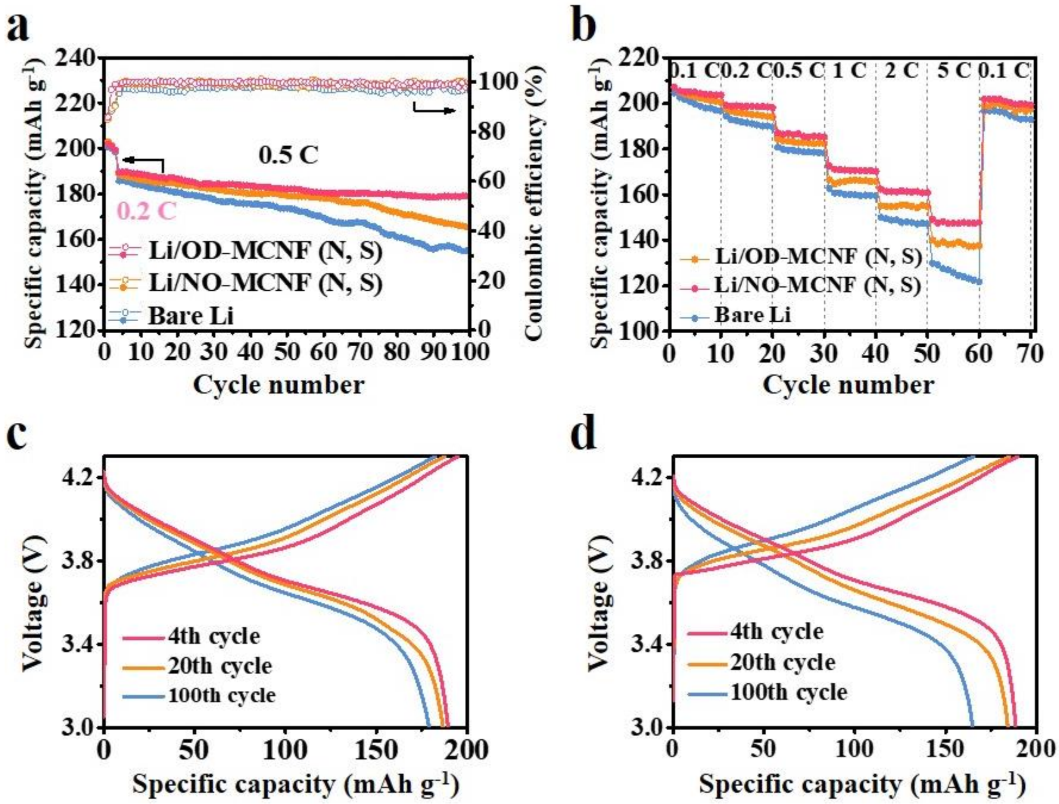

Fig. S29. **a**, Long-term cycling performances and **b**, rate performances of full coin cells constituted of various anodes with NCM811 cathode with the voltage ranging from 3.0 to 4.3 V. The charge/discharge curves of **c**, Li/OD-MCNF (N, S)||NCM811 and **d**, Li/NO-MCNF (N, S)||NCM811 full coin cells at 0.2 C with the voltage ranging from 3.0 to 4.3 V.

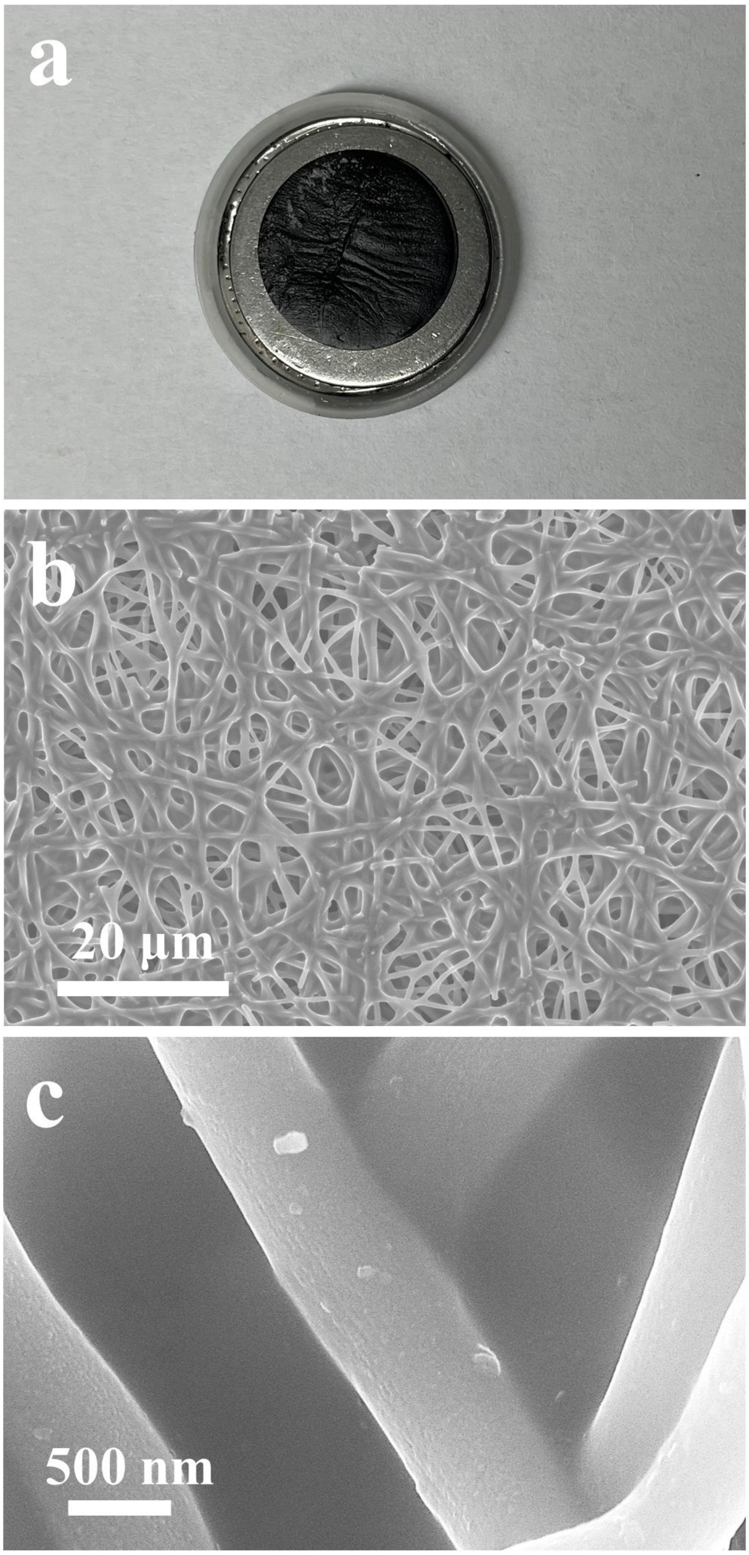

Fig. S30. a, The digital photograph and **b, c**, SEM images of the Li/OD-MCNF (N, S) anode in the Li/OD-MCNF (N, S)||NCM811 full coin cell after 100 cycles at 0.2 C with the voltage ranging from 3.0 to 4.3 V. The Li/OD-MCNF (N, S) anode keep intact and no obvious surface cracks can be observed after cycling. Moreover, the mesoporous structure reserved. It is clear that the OD-MCNF (N, S) has outstanding cycling stability.

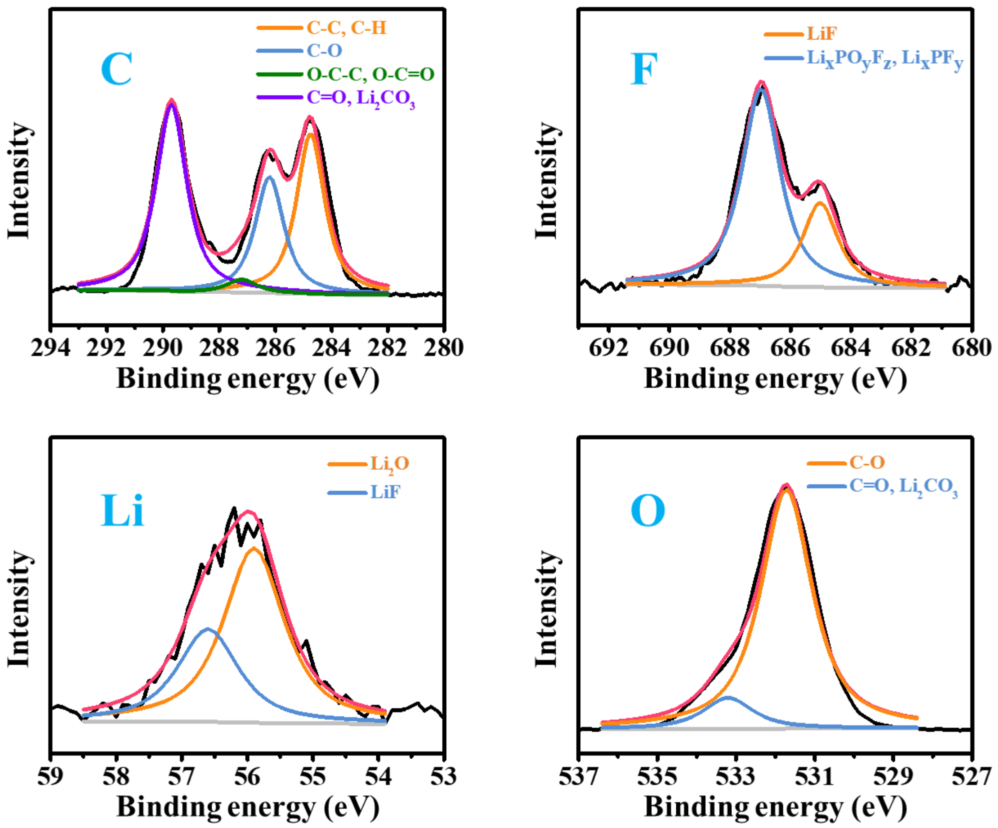

Fig. S31. The XPS spectra of Li/OD-MCNF (N, S) anode in the Li/OD-MCNF (N, S)||NCM811 full coin cell after 100 cycles at 0.2 C with the voltage ranging from 3.0 to 4.3 V: **a**, C 1s, **b**, F 1s, (**c**) Li 1s and **d**, O 1s spectra. The formation of LiF/$Li_xPO_yF_z$ in the SEI layer likely contributed to the enhanced cycling [4].

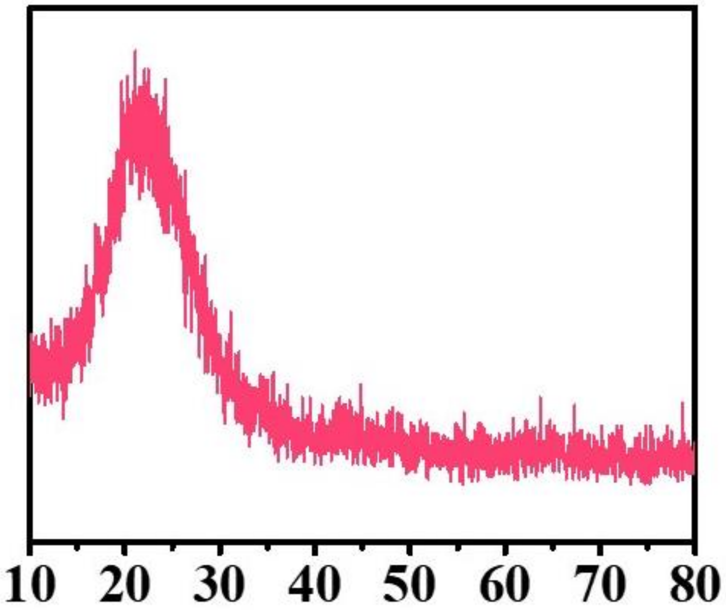

Fig. S32. XRD pattern of the Li/OD-MCNF (N, S) anode in the Li/OD-MCNF (N, S)||NCM811 full coin cell after 100 cyclest 0.2 C with the voltage ranging from 3.0 to 4.3 V. The peak intensity decreased compared to the OD-MCNF (N, S) (Supplementary Fig. S6), caused by shadow effect of the SEI film.

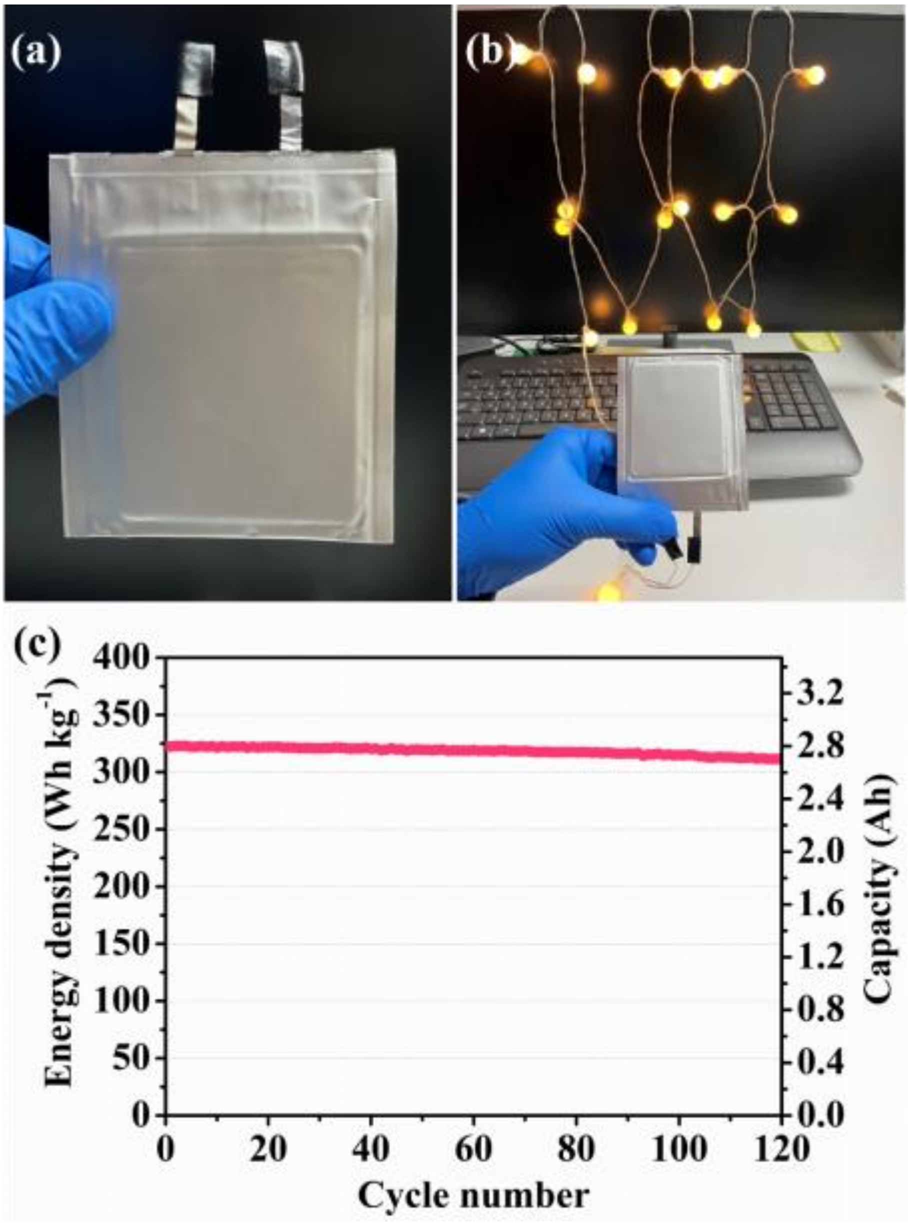

Fig. S33. a, b, The optical photographs of Li/OD-MCNF (N, S)||NMC811 pouch cell. **c**, The cycling performance of the Li/OD-MCNF (N, S)||NMC811 pouch cell at 0.1 C. Note that the energy density is calculated using the total weight of the whole cell.

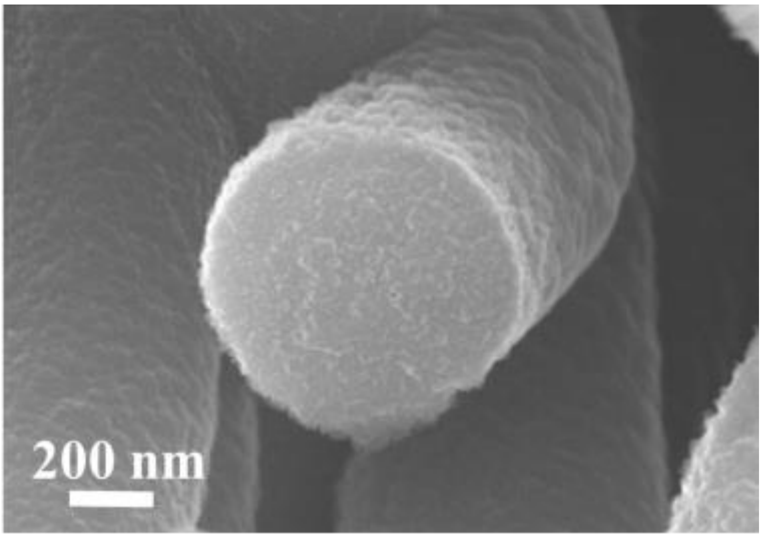

Fig. S34. SEM image of cross-section of the OD-MCNF (N, S) with full lithiation.

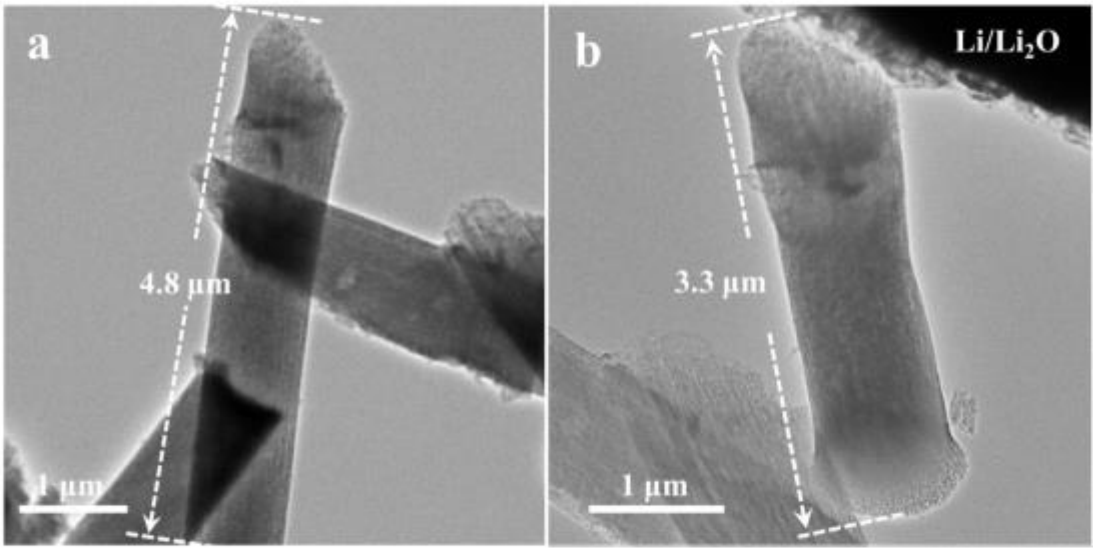

Fig. S35. a, b, *In-situ* TEM images of OD-MCNF (N, S) before and after contacting with the Li/Li_2O electrode. The OD-MCNF (N, S) was fractured by excessive lithium plating.

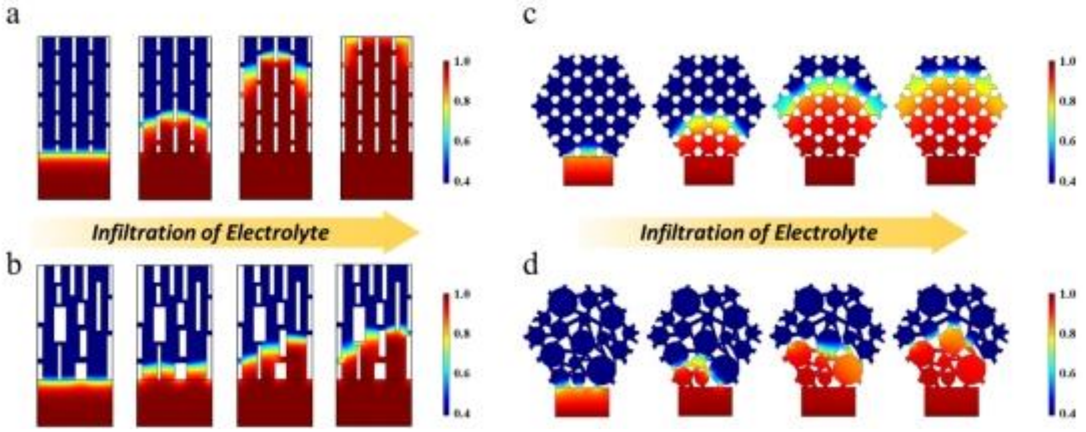

Fig. S36. The simulation results of the transfer of electrolyte in **a**, planar and **b**, cross-sectional for both the OD-MCNF (N, S) and NO-MCNF (N, S) using COMSOL Multiphysics.

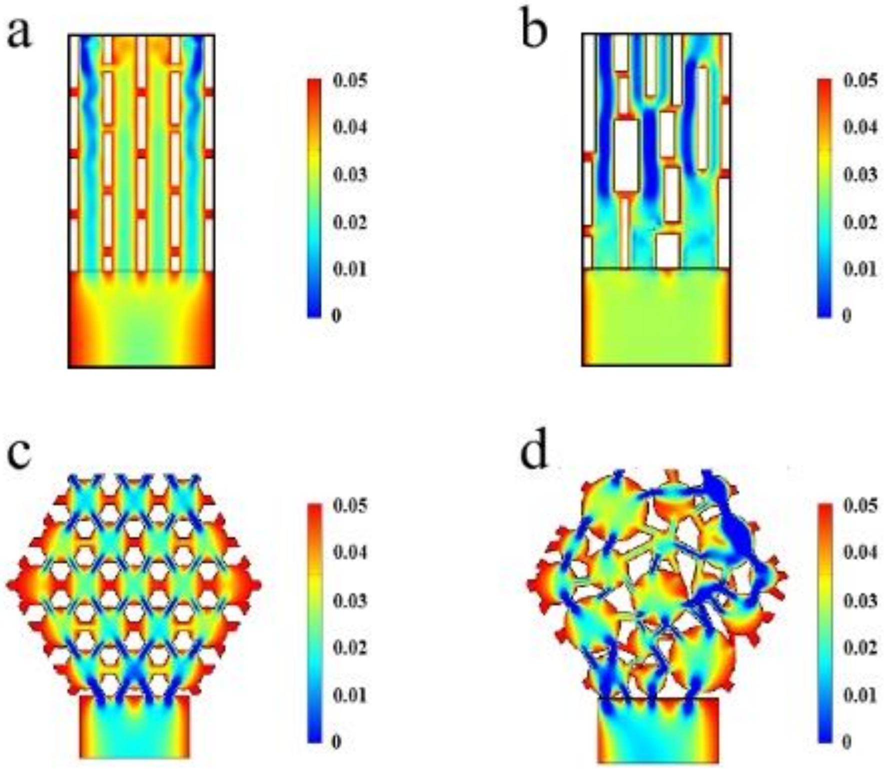

Fig. S37. COMSOL simulation of the transfer rate of electrolyte in different models.

Supplementary Tables

Table S1. Texture parameters of the different samples.

Samples	S_{BET} (m^2 g^{-1})	V_t (cm^3 g^{-1})	V_{mi} (cm^3 g^{-1})	V_{me} (cm^3 g^{-1})	Pore size (nm)
OD-MCNF-5	545	0.71	0.15	0.56	15.1
OD-MCNF-10	531	0.77	0.14	0.63	17.8
OD-MCNF-15	462	0.78	0.14	0.64	20.4
OD-SiO_2MNF	360	0.75	0.10	0.65	21.9
OD-TiO_2 MNF	126	0.40	-	0.40	22.1
OD-WO_3 MNF	49	0.22	-	0.22	25.9
OD-MCNF (N, S)	766	0.82	0.21	0.61	1.0, 20.0
NO-MCNF (N, S)	707	0.65	0.32	0.33	0.9, -

Notation: S_{BET}, BET surface area; V_t, total pore volume; V_{mi}, volume of micropores and V_{me}, volume of mesopores.

Table S2. The content of different elements in OD-MCNF (N, S) and NO-MCNF (N, S).

Samples	C_{1s} (at.%)	N_{1s} (at.%)	O_{1s} (at.%)	S_{2p} (at.%)
OD-MCNF (N, S)	90.8	4.1	3.4	1.7
NO-MCNF (N, S)	92.6	3.0	3.3	1.1

Table S3. The content of different N and S species present in OD-MCNF (N, S) and NO-MCNF (N, S).

Samples	N_{1s} (%)				S_{2p} (%)	
	Pyridinic-N	Pyrrolic-N	Graphitic-N	Oxidized-N	Thiophene-S_{2p}	C-SO_x-C
OD-MCNF (N, S)	62.2	20.5	12.9	5.4	68.4	31.6
NO-MCNF (N, S)	55.0	28.3	11.7	6.3	75.5	24.5

Table S4 Comparison of electrochemical properties of symmetrical cells.

	Materials	Combining method with Li	Current density (mA cm^{-2})/ Cycling capacity (mAh cm^{-2})	Voltage polarization (mV)	Maximum cycle number (time)	Rate current density (mA cm^{-2})/Cycling capacity (mAh cm^{-2})	Voltage polarization (mV)	Title	Periodical	Year of publication	Reference
1	N-doped amorphous Zn-carbon multichannel fibers decorated with carbon cages (CC-Zn-CMFs)	Eletroplating	1/1	~30 (2000 h)	2000 h	0.5/2	~15	Nitrogen-Doped Amorphous Zn-Carbon Multichannel Fibers for Stable Lithium Metal Anodes	Angewandte Chemie International Edition	2021	[5]
						1/2	~19				
						2/2	~31				
						3/2	~39				
						5/2	~62				
						10/2	~139				
2	Li-coated CNT sponge macrofilm （LiCSMF）	Infusion of molten Li into CNT sponge macrofilm	2/1	12 (300th cycle)	300	10/10	~53	A Dendrite-Free Lithium/Carbon Nanotube Hybrid for Lithium-Metal Batteries	Advanced Materials	2020	[6]
			20/10	67.5 (300th cycle)	300	20/10	~67				
			40/2	54 (1900th cycle)	2000	30/10	~80				
						40/10	~107				
3	Cellulose/graphene carbon composite aerogel (CCA)	Placing a layer of CCA on the bottom of the Li metal	10/1	118 (1000th cycle)	1000	1/1	23	Current-Density Regulating Lithium Metal Directional Deposition for Long Cycle-Life Li Metal Batteries	Angewandte Chemie International Edition	2021	[7]
			1/4	~20	2000 h	2/1	~26				
			25/13	~50	300 h	3/1	~43.5				
						5/1	~52				
						10/1	~87				

						20/1	~96				
						30/1	110				
4	Functional grafting of superstructure single-ion conducting polymer on cellulose nanofibril (CNF-g-PSSLi)	Coating CNF-g-PSSLi membrane on Li metal	1/1	21 (900 h)	900 h	1/20	~71	A polymer brush-based robust and flexible single-ion conducting artificial SEI film for fast charging lithium metal batteries	Energy Storage Materials	2021	[8]
						5/20	~244				
			5/1	19 (1000 h)	1000 h	10/20	~112				
			20/20	~118 （270 h）	270 h	20/20	~118				
5	Dual-gradient 3D silver nanowires (AgNWs)/CNT scaffold	Eletroplating	10/1	~74	800	0.5/0.1	~11	Ultralow-Expansion Lithium Metal Composite Anode via Gradient Framework Design	Advanced Functional Materials	2022	[9]
			30/1	~117	500	1/0.2	~13				
			40/1	~164	400	5/1	~26				
						10/2	~31				

						20/4	~48				
6	ZnO-multi-level Cu nanofibers on a Cu foam (ZnO-MCNCF)	Eletroplating	1/1	~11 (900 h)	900 h	0.5/1	~8	Atomic layer deposition assisted superassembly of ultrathin ZnO layer decorated hierarchical Cu foam for stable lithium metal anode	Energy Storage Materials	2021	[10]
			3/1	23 (275 h)	275 h	1/1	~14				
			5/10	23.9 (3000 h)	3000 h	2/1	~28				
			10/10	57.1 (1400 h)	6	4/1	~38				
						8/1	~67				
7	Carbon-coated mixed metal fluoride (NMMF@C) core@shell microparticles	Eletroplating	1/3	20	1600	-	-	An ultrastable lithium metal anode enabled by designed metal fluoride spansules	Science Advances	2020	[11]

8	2D mesoporous polypyrrole-graphene oxide (mPPy-GO)	Eletroplating	1/1	9 (1100 h)	1100 h	0.25/1	4	A Two-Dimensional Mesoporous Polypyrrole-Graphene Oxide Heterostructure as a Dual-Functional Ion Redistributor for Dendrite-Free Lithium Metal Anodes	Angewandte Chemie International Edition	2020	[12]
						0.5/1	~13				
						1/1	~16				
			5/1	22 (400 h)	400 h	3/1	~25				
						5/1	~29				
						10/1	35				
9	Nitrogen, sulfur codoped carbon dots (N, S-CDs)	Eletroplating	0.5/1	~27 (200th cycle)	200	0.5/1	~27	N, S-codoped carbon dots as deposition regulating electrolyte additive for stable lithium metal anode	Energy Storage Materials	2021	[13]
						1/1	~28				
			1/1	~23 (1200 h)	1200 h (600 cycles)	2/1	~36				
						3/1	~46				
			3/1	~75 (300 h)	300 h (450 cycles)	5/1	~66				
10	A thin layer of PIL containing	Coating PDDA-TFSI on	1/1	~23 (1000 h)	1000 h	0.5/0.5	~32	Polycationic Polymer Layer for	Adcanced Materials	2021	[14]

	polymeric cation of poly(diallyl dimethyl ammonium) and bis(trifluoromethane sulfonyl)imide anion (PDDA-TFSI)	Li metal				1/1	~40	Air-Stable and Dendrite-Free Li Metal Anodes in Carbonate Electrolytes			
			10/1	~63 (700 h)	700 h	2/2	~45				
						3/3	~59				
			5/5	~228 (~70 h)	~70 h	4/4	~76				
						5/5	~96				
11	OD-MCNF (N, S)	Infusion of molten Li into OD-MCNF (N, S)	2/1	~5 (3000 h)	3000 h	1/1	1				This work
						2/1	3.4				
						3/1	3.7				
						4/1	6.2				
						5/1	8				
						10/1	15				
						20/1	21				
						30/1	28				

Table S5. Standardized data reporting for coin batteries.

**Battery assembly reporting**	**Explain exception if line item is not present in manuscript**
Ratio or mass percent of active material, conductive additive, and binder on electrode	NCM811 cathode: 92 wt% NCM811, 3 wt% of Super-p, and 5 wt% of PVDF binder<br>$LiFePO_4$ cathode: 80 wt% NCM811, 10 wt% of Super-p, and 10 wt% of PVDF binder
Amount of electrolyte (either volume or mass) used as well as electrolyte components by percent composition	Half cells and symmetric cells: The electrolyte contained 1 M LiTFSI in a mixture of DOL and DME (1:1 in volume) with 1 wt.% $LiNO_3$ as an additive.<br>Full cells: 1.0 M $LiPF_6$ in ethylene carbonate and diethyl carbonate (v:v = 1:1) for full cell.<br>About 100 μL per coin cell.
Areal mass (mg/cm^2) loading of active material on current collector	NCM811 cathode: ~18 mg cm^{-2}<br>$LiFePO_4$ cathode: ~8 mg cm^{-2}
For Li metal cells, the amount of excess lithium being used (thickness and capacity of the excess foil)	0.4 mg Li per anode
**Electrochemical reporting**	
Temperature at which electrochemical tests are run	25 °C
Indicate whether full-cells or half-cells are used for electrochemical tests.	Full-cells and half-cells.
First cycle or any pre-cycling electrochemical data	The pre-cycling electrochemical data has been shown in the article.
Volumetric and gravimetric stack energy densities with calculation methodology	Calculated by the content of active materials of cathode.
Theoretical capacity used to calculate C-rate	NCM811 full cells: 200 mAh g^{-1}<br>$LiFePO_4$ full cells: 170 mAh g^{-1}
C-rate clearly reported on the figure or in the figure caption for every electrochemical test	NCM811 full cells: 0.1, 0.2, 0.5, 1, 2 and 5 C<br>$LiFePO_4$ full cells: 0.2, 0.5, 1, 2 and 5 C

Table S6. The parameters of the Li/OD-MCNF (N, S)||NMC811 pouch cell at the 2.8 Ah level.

	Parameter	Value
NCM811 cathode	Discharge capacity	190 mAh g^{-1}
	Area weight (each side)	29.26 mg cm^{-2}
	Area capacity (each side)	5.56 mAh cm^{-2}
	Number of layers	6
Li anode (Li:C is 2.8:1)	Areal capacity	7.61 mAh cm^{-2} (~100 μm thick)
	Area weight	2.44 mg cm^{-1}
	Number of layers	7
Al foil	Thickness	12 μm
Electrolyte	E/C ratio	3 g Ah^{-1}
Separator	Thickness	20 μm
Package foil	Thickness	115 μm
Cell	Average voltage	3.65 V
	Total weight	30 g
	Energy density	323 Wh kg^{-1}

Reference

1. Wang CY, Zhao YJ, Zhou LL *et al.* Mesoporous carbon matrix confinement synthesis of ultrasmall WO_3 nanocrystals for lithium ion batteries. *J Mater Chem A* 2018; **6**: 21550-7.

2. Luo W, Zhao T, Li YH *et al.* A micelle fusion-aggregation assembly approach to mesoporous carbon materials with rich active sites for ultrasensitive ammonia sensing. *J Am Chem Soc* 2016; **138**: 12586-95.

3. Fang C, Li J, Zhang M et al. Quantifying inactive lithium in lithium metal batteries. *Nature* 2019; **572**: 511-5.

4. Arnot DJ, Li W, Bock DC et al. Low-Oxidized Siloxene Nanosheets with High Capacity, Capacity Retention, and Rate Capability in Lithium-Based Batteries. *Adv Mater* 2022; **9**: 2102238.

5. Fang Y, Zeng Y, Jin Q et al. Nitrogen-doped amorphous Zn-carbon multichannel fibers for stable lithium metal anodes. *Angew Chem Int Ed* 2021; **60**: 8515-8520.

6. Wang ZY, Lu ZX, Guo W et al. A dendrite-free lithium/carbon nanotube hybrid for lithium-metal batteries, *Adv Mater* 2021; **33**: 2006702.

7. Mao H, Yu W, Cai Z et al. Current-Density Regulating Lithium Metal Directional Deposition for Long Cycle-Life Li Metal Batteries. *Angew Chem Int Ed* 2021; **60**: 19306-19313.

8. Zeng J, Liu Q, Jia D et al. A polymer brush-based robust and flexible single-ion conducting artificial SEI film for fast charging lithium metal batteries. *Energy Stor Mater* 2021; **41**: 697-702.

9. Liu YC, Yuan BY, Sun C et al. Ultralow-expansion lithium metal composite anode via gradient framework design. *Adv Funct Mater* 2022; **32**: 2202771.

10. Zhang RH, Li Y, Qiao L et al. Atomic layer deposition assisted superassembly of ultrathin ZnO layer decorated hierarchical Cu foam for stable lithium metal anode. *Energy Stor Mater* 2021; **37**: 123-34.

11. Yuan H, Nai J, Tian H et al. An ultrastable lithium metal anode enabled by designed metal fluoride spansules. *Sci Adv* 2021; **6**: 3112.

12. Shi H., Qin J, Huang K et al. A two-dimensional mesoporous polypyrrole-graphene oxide heterostructure as a dual-functional ion redistributor for dendrite-free lithium metal anodes. *Angew Chem Int Ed* 2020; **59**: 12147-53.

13. Li S, Luo Z, Tu H et al. S-codoped carbon dots as deposition regulating electrolyte additive for stable lithium metal anode. *Energy Stor Mater* 2021; **42**: 679-86.

14. Wu J, Rao Z, Liu X et al. Polycationic polymer layer for air-stable and dendrite-free Li metal anodes in carbonate electrolytes. *Adv Mater* 2021; **33**: 2007428.
